# Supplementary material for: Proteomic and Single-Cell Transcriptomic Dissection of Human Plasmacytoid Dendritic Cell Response to Influenza Virus
Source: Front Immunol. 2022 Mar 23;13:814627. doi: 10.3389/fimmu.2022.814627 (PMC8984281; doi:10.3389/fimmu.2022.814627)
Supplement: Supplementary file 1 [file DataSheet_1.pdf]

Supplementary materials for Ghanem MH et al

Proteomic and single-cell transcriptomic dissection of human plasmacytoid dendritic cell response to influenza virus

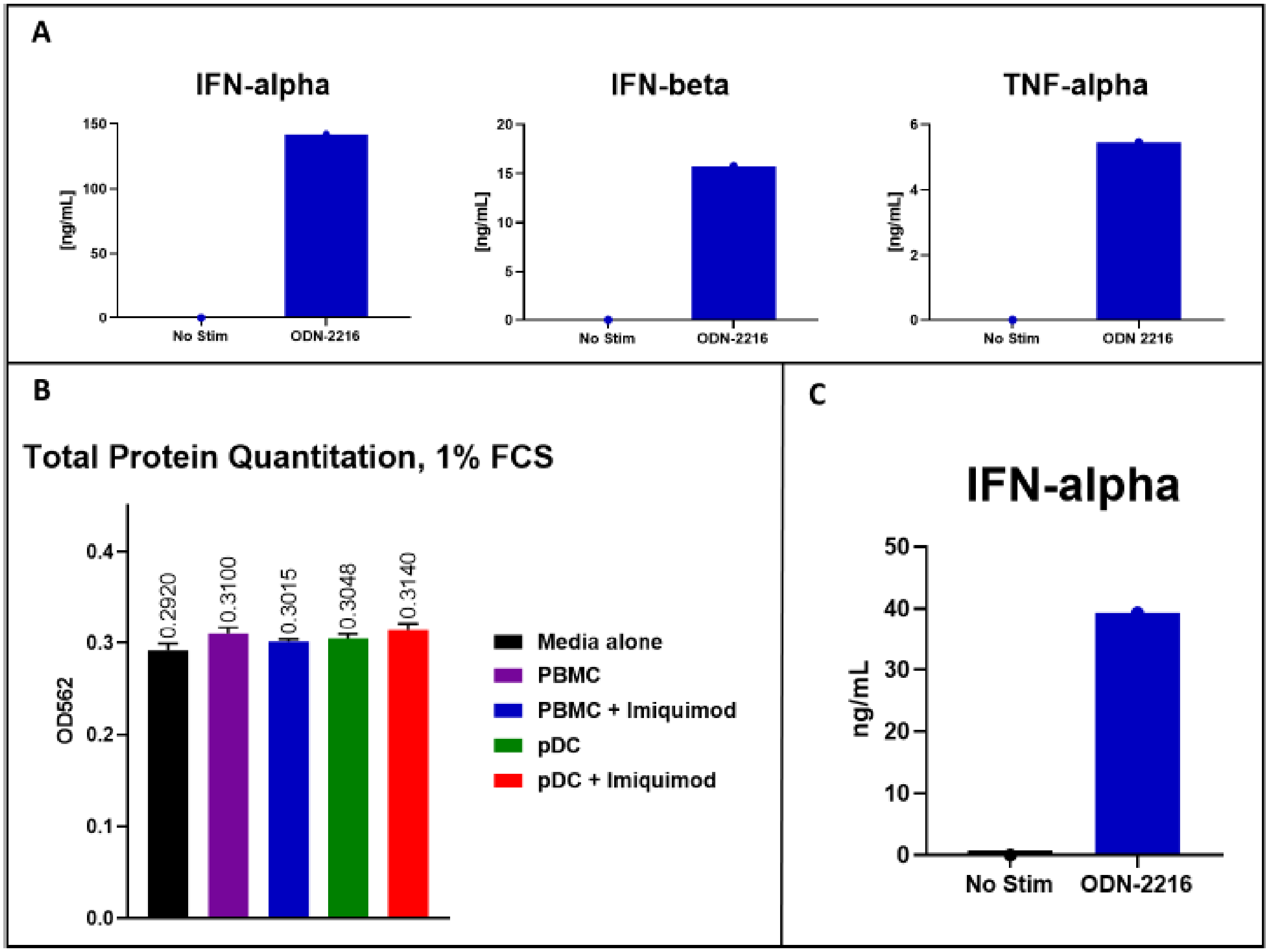

Figure S1: Media optimization towards LC-MS/MS.

A) pDCs cultured in the presence of 1% FCS are highly responsive to stimulation, producing robust amounts of IFN-alpha, IFN-beta and TNF-alpha. B) BCA assay for total protein content. Despite substantial cytokine production, pDCs only marginally enrich the protein content of the culture media, as >90% of media protein is derived from FCS alone. Stimulated pDCs were no different in this regard than unstimulated pDCs or PBMCs. C) pDCs cultured in Advanced RPMI, containing minimal amounts of albumin, insulin and holo-transferrin, without any serum supplement, remain highly functional. ODN2216, TLR9 agonist. Imiquimod, TLR7 agonist.

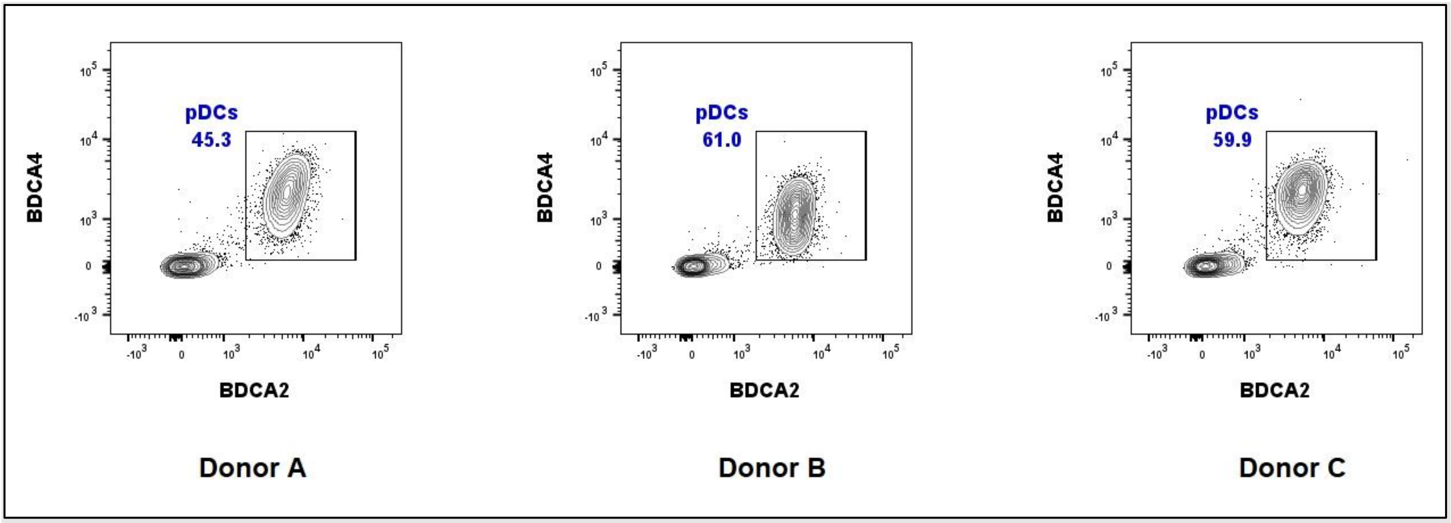

Figure S2: pDC purity for the secretome cultures.

pDCs were enriched from 3 donors emphasizing yield over purity. Purity was determined by staining for BDCA2 and BDCA4. Cell yields were: Donor A, 4.2e6; Donor B 3.7e6, Donor C 1.5e6 total cells. Cell viability was >95% by fluorescent cell counting using Acridine Orange / Propidium Iodide staining and the Cellometer Auto 2000 instrument. Pre-enrichment pDC frequency was 0.2-0.3% among PBMCs in all donors.

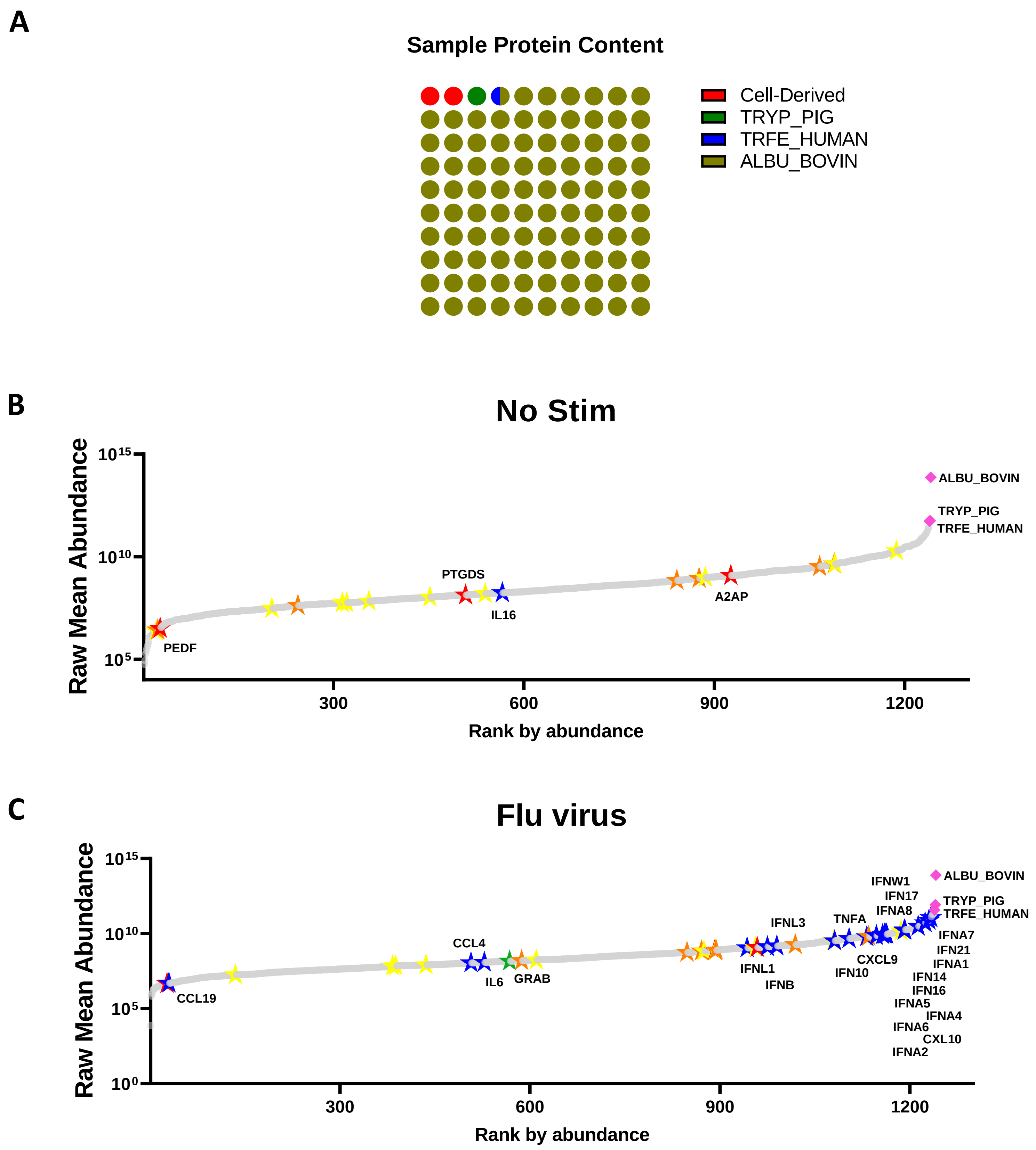
A
Sample Protein Content
Cell-Derived
TRYP_PIG
TRFE_HUMAN
ALBU_BOVIN
B
No Stim
Raw Mean Abundance
10^{15}
10^{10}
10^{5}
ALBU_BOVIN
TRYP_PIG
TRFE_HUMAN
PTGDS
IL16
A2AP
PEDF
300
600
900
1200
Rank by abundance
C
Flu virus
Raw Mean Abundance
10^{15}
10^{10}
10^{5}
10^{0}
ALBU_BOVIN
TRYP_PIG
TRFE_HUMAN
IFNW1
IFN17
IFNA8
IFNL3
TNFA
CCL4
IL6
GRAB
CCL19
IFNL1
IFNB
CXCL9
IFN10
IFNA7
IFN21
IFNA1
IFN14
IFN16
IFNA5
IFNA4
IFNA6
CXL10
IFNA2
300
600
900
1200
Rank by abundance

Figure S3. The pDC baseline and influenza-induced secretomes.

A) Protein content in the samples by relative abundance. B) Proteins identified in the unstimulated group ranked by average abundance. C) Proteins identified in the Influenza H1N1-stimulated group ranked by average abundance. For B and C: Data points represent unique proteins. Pink diamonds; exogenous proteins. Yellow, orange, red stars: proteins annotated as ‘secreted’ in the UniProt database, with relative enrichment of expression in pDCs among circulating leukocytes according to the Protein Atlas (http://www.proteinatlas.org/), with yellow=mild, orange=moderate, and red=significant pDC enrichment. Blue stars; cytokines/chemokines. Green star; granzyme B. Gray data points, with all points fused into a single line, represent proteins not annotated as ‘secreted’ in UniProt or not significantly enriched for expression in pDCs according to the Protein Atlas. Select proteins of interest are directly labeled.

A

HEMO_HUMAN, Hemopexin

Example Peptide Abundances

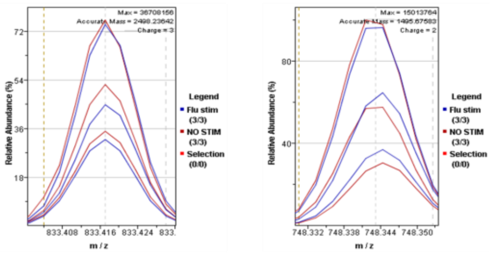

EVGTPHGIILDSVDAA FIC[160.0307]PGSSR (3+)

YYC[160.0307]FQGNQFLR (2+)

Peptide Count	Mean Ion Score	Mean Protein Score	Ratio (Flu stim/ NO STIM)	P-value (Flu stim vs. NO STIM)
20	57	1,133	0.99	0.89

HEMO_HUMAN, Hemopexin

Example MS/MS Spectra

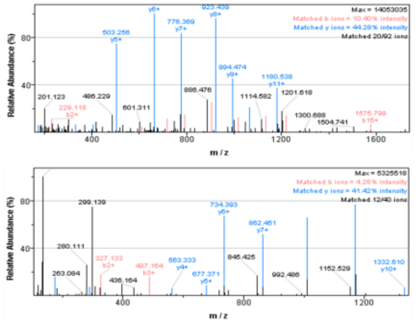

EVGTPHGIILDSVDAA FIC[160.0307]PGSSR (3+)

YYC[160.0307]F QGNQFLR (2+)

B

DPYL2_HUMAN, Dihydropyrimidinase-related protein 2

Example Peptide Abundances

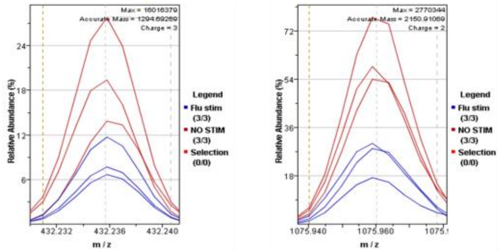

MVIPGGIDVHTR (3+)

FQMPDQGMTSADDFFQGTK (2+)

Peptide Count	Mean Ion Score	Mean Protein Score	Ratio (Flu stim/ NO STIM)	P-value (Flu stim vs. NO STIM)
21	61	1,065	0.46	3.4E-27

DPYL2_HUMAN, Dihydropyrimidinase-related protein 2

Example MS/MS Spectra

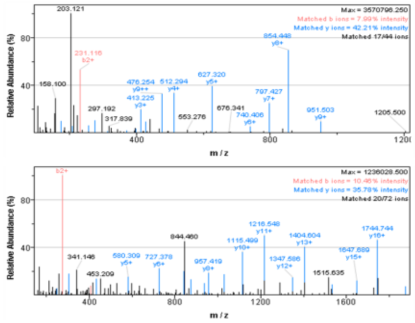

MVIPGGIDVHTR (3+)

FQMPDQGMTSA DDFFQGTK (2+)

C

TNFA_HUMAN, Tumor necrosis factor

Example Peptide Abundances

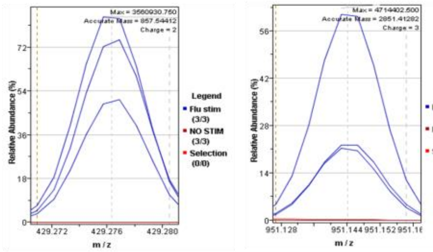

VNLLSAIK (2+)

ETPEGAEAKPWYEPIYL GGVFQLEK (3+)

Peptide Count	Mean Ion Score	Mean Protein Score	Ratio (Flu stim/ NO STIM)	P-value (Flu stim vs. NO STIM)
5	38	137	72.1	3.5E-17

TNFA_HUMAN, Tumor necrosis factor

Example MS/MS Spectra

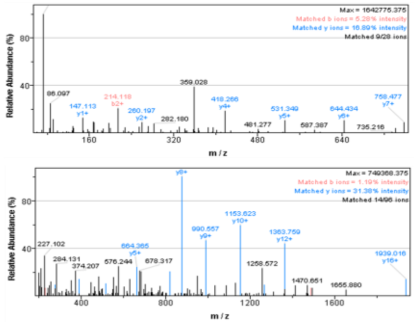

VNLLSAIK (2+)

ETPEGAEAKPWYEPIYL GGVFQLEK (3+)

D

IFN21_HUMAN, Interferon alpha-21

Example Peptide Abundances

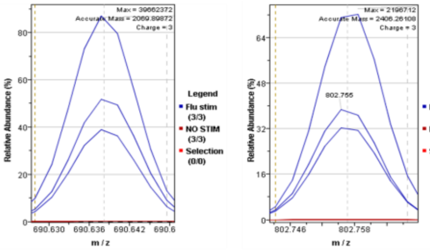

HDFGFPQEEFDG NQFQK (3+)

AQAISVLHEMIQ QTFNLFSTK (3+)

Peptide Count	Mean Ion Score	Mean Protein Score	Ratio (Flu stim/ NO STIM)	P-value (Flu stim vs. NO STIM)
7	64	1,432	468	2.0E-21

IFN21_HUMAN, Interferon alpha-21

Example MS/MS Spectra

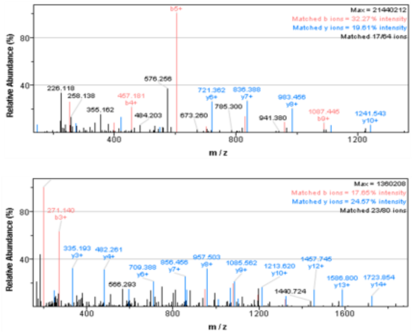

HDFGFPQEEFDG NQFQK (3+)

AQAISVLHEMIQ QTFNLFSTK (3+)

Figure S4. Representative protein species identified in the baseline and induced pDC secretomes.

For each protein, example graphics for two peptides are shown, including a monoisotopic MS peak (feature plotted as relative % abundance) as generated by the Elucidator program (charge state indicated), and an ms/ms spectrum for each peptide. Blue peaks are y-ions, red peaks are b-ions, and black are unmatched peaks.The feature peaks (left) in each case are an overlay of aligned monoisotopic peaks of MS spectra for the 12 LC-MS/MS runs in the experiment. Below the MS feature graphics is a table representing quantitation and identification details for each protein. A. Hemopexin, a protein produced by the liver and not known to be produced by circulating leukocytes, is not significantly different in preparations from unstimulated and flu stimulated cells. B. DPYL2, a metabolic enzyme, is enriched in the media of unstimulated cells compared to flu stimulated cells. C-D. Interferon α-1/13 and TNF-α are tremendously upregulated in the flu stimulated condition.

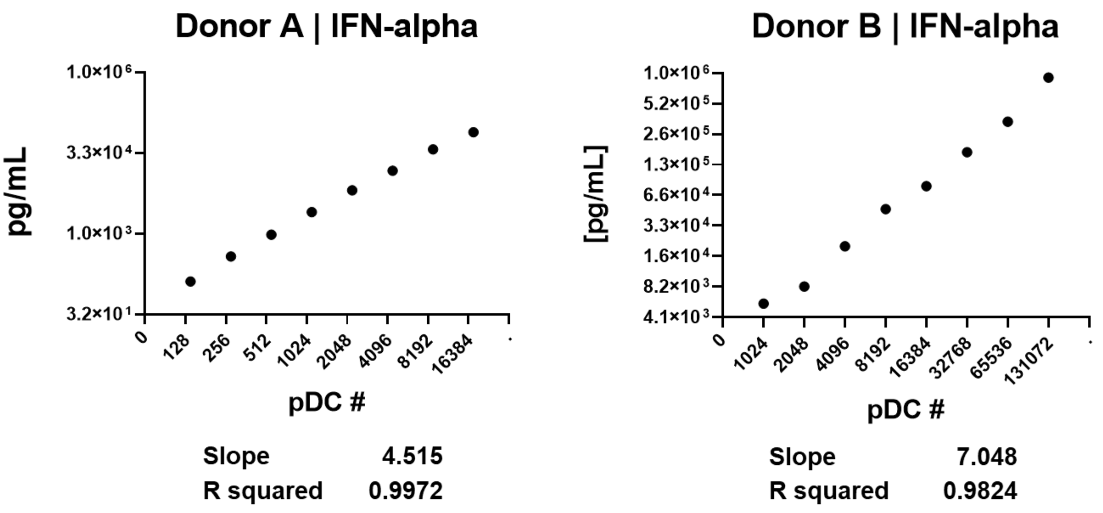

Figure S5. Relationship between pDC input in IFN-α output using influenza H1N1 as the stimulus.

Data derived from pDCs cultured for 24hrs in 96-well plates, with cells in a fixed 200uL culture volume per well. IFN-alpha levels determined using pan-subtype ELISA.

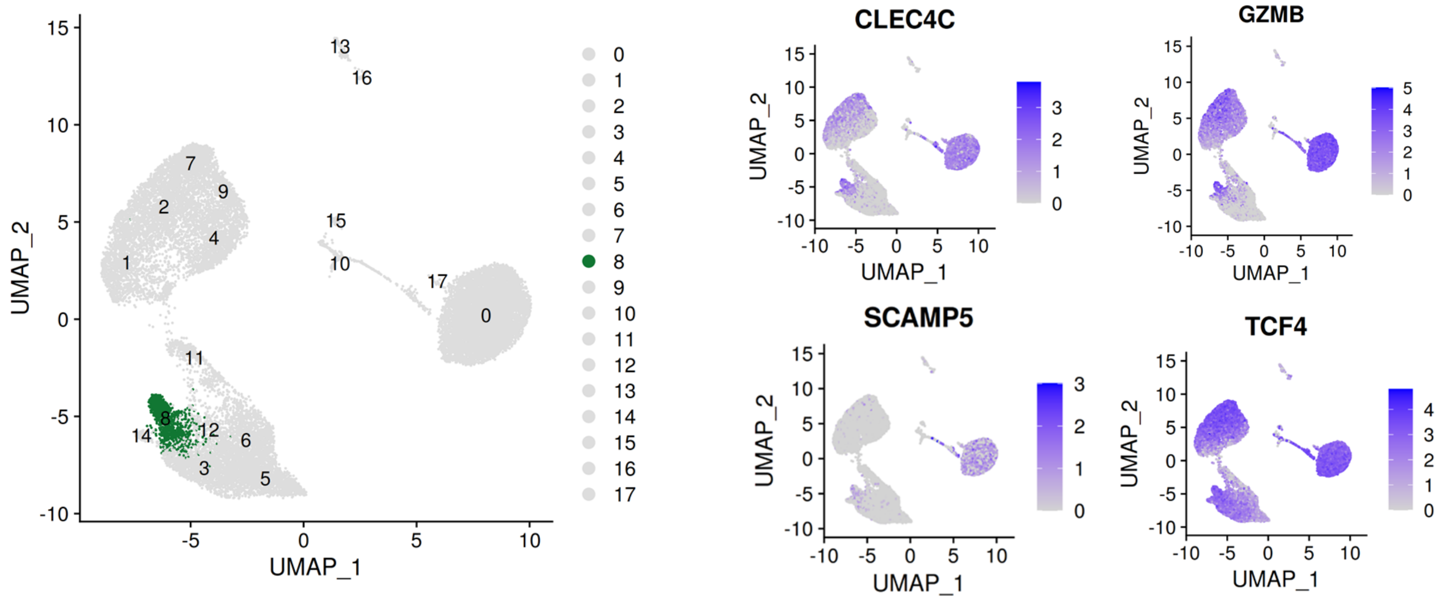

Figure S6. Cluster 8 retains features of unperturbed pDCs to 24hrs in the presence of influenza virus.

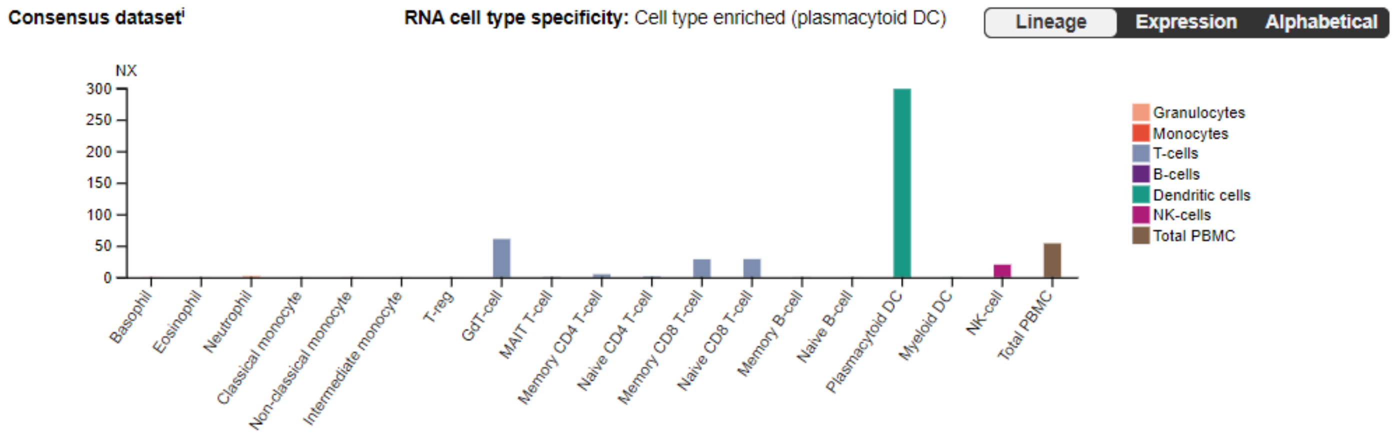

Figure S7. Expression of Granzyme B across leukocyte subsets in the Human Protein Atlas.

Table S1 LC-MS/MS Identified Proteins in the pDC Secretome with normalized expression data.

UniProt ID	Peptide Count	Ratio (Flu stim:NO STIM)	P-value (Flu stim vs. NO STIM)	-LOG(P)
IFN10	2	306	0	>40
IFN17	2	91.6	0	>40
IFNW1	3	44.2	0	>40
IFNA8	4	901	0	>40
IFNA7	10	470	0	>40
IFNB	3	647	3.6E-40	39.4432
IFN14	4	508	1.0E-33	33.0001
IFNA4	4	550	2.3E-30	29.6356
IFNA6	5	234	1.1E-28	27.9531
IFN16	6	94.9	3.1E-28	27.5147
DPYL2	21	0.46	3.4E-27	26.472
IFNA5	2	56.1	1.3E-25	24.8755
PNCB	4	0.36	9.5E-25	24.0244
IFNL3	2	186	2.0E-23	22.71
IFN21	7	468	2.0E-21	20.6942
IFNA2	4	296	2.2E-18	17.6657
TNFA	5	72.1	3.5E-17	16.4548
CALU	1	8.21	1.1E-16	15.9626
IFNA1	10	499	4.6E-16	15.3368
H2AY	12	0.44	2.6E-13	12.5894
CCL4	1	6.85	6.5E-13	12.1896
COR1B	5	0.43	3.4E-12	11.4641
H1X	5	0.28	1.4E-10	9.86614
LGMN	4	11.0	1.8E-10	9.7428
PDCD4	2	0.14	5.3E-10	9.27597
TCL1A	2	0.49	8.8E-10	9.05453
GLO2	2	0.35	4.4E-09	8.36002
IFIT3	7	40.5	8.1E-09	8.09334
HP1B3	2	0.24	1.1E-08	7.96098
RHG17	6	0.48	5.2E-08	7.28802
COR1C	17	0.39	9.2E-08	7.03763
MPRD	2	0.41	2.9E-07	6.53745
CXL10	6	118	4.7E-07	6.32873
PACN1	12	0.41	4.8E-07	6.31623
FSCN1	12	11.0	6.1E-07	6.21789
PRP19	6	0.41	7.6E-07	6.11827

**PAF1**	**1**	**0.26**	**8.5E-07**	**6.07109**
**VAPA**	**2**	**0.35**	**8.7E-07**	**6.06053**
**VP13C**	**6**	**0.38**	**1.1E-06**	**5.95468**
**FLNB**	**56**	**0.44**	**1.5E-06**	**5.81446**
**VATB2**	**6**	**0.51**	**2.6E-06**	**5.58603**
**SC22B**	**3**	**0.23**	**2.6E-06**	**5.57873**
**CNDP2**	**21**	**0.46**	**2.8E-06**	**5.55705**
**RAB7A**	**5**	**0.36**	**3.5E-06**	**5.45817**
**CATB**	**5**	**16.1**	**4.6E-06**	**5.34008**
**LMNB2**	**15**	**0.36**	**5.0E-06**	**5.29722**
**RB11B**	**8**	**0.33**	**6.0E-06**	**5.21961**
**CATC**	**14**	**33.0**	**6.3E-06**	**5.19921**
**CLCA**	**1**	**0.40**	**6.7E-06**	**5.17594**
**H15**	**8**	**0.46**	**9.7E-06**	**5.01341**
**SC24C**	**9**	**0.42**	**1.1E-05**	**4.94348**
**RUXE**	**2**	**0.28**	**1.3E-05**	**4.87484**
**VATG1**	**3**	**0.53**	**1.4E-05**	**4.84285**
**U520**	**6**	**0.36**	**1.5E-05**	**4.83239**
**ROCK1**	**1**	**0.49**	**1.5E-05**	**4.81531**
**RU17**	**6**	**0.40**	**1.7E-05**	**4.76246**
**ISG20**	**2**	**4.84**	**1.7E-05**	**4.7602**
**PAXX**	**4**	**0.38**	**1.9E-05**	**4.71897**
**H14**	**8**	**0.40**	**2.1E-05**	**4.66979**
**RET4**	**5**	**0.73**	**2.4E-05**	**4.62599**
**HNRL2**	**7**	**0.45**	**3.5E-05**	**4.45556**
**IFNL1**	**3**	**31.7**	**3.5E-05**	**4.45432**
**ARHG7**	**3**	**0.32**	**3.9E-05**	**4.40506**
**FA49B**	**14**	**0.45**	**4.3E-05**	**4.36917**
**IC1**	**4**	**18.3**	**4.7E-05**	**4.33106**
**CAB45**	**3**	**6.61**	**5.2E-05**	**4.28108**
**SRRT**	**1**	**0.40**	**5.9E-05**	**4.22775**
**KTN1**	**3**	**0.34**	**6.8E-05**	**4.16577**
**CAPG**	**14**	**0.50**	**6.9E-05**	**4.15889**
**HDAC2**	**3**	**0.27**	**7.0E-05**	**4.15783**
**H12**	**1**	**0.43**	**8.1E-05**	**4.0913**
**ZC3HF**	**1**	**0.39**	**8.8E-05**	**4.05596**
**LGUL**	**1**	**0.33**	**9.8E-05**	**4.00997**
**TLN1**	**85**	**0.50**	**9.8E-05**	**4.00948**
**VPS25**	**1**	**0.22**	**1.0E-04**	**3.99396**
**HNRL1**	**1**	**0.36**	**1.0E-04**	**3.97964**
**NUMA1**	**15**	**0.34**	**1.1E-04**	**3.95195**
**HPLN3**	**5**	**43.5**	**1.1E-04**	**3.94539**
**STAT1**	**14**	**7.51**	**1.2E-04**	**3.9333**
**IFIT2**	**5**	**46.5**	**1.4E-04**	**3.84802**

RBM25	2	0.38	1.4E-04	3.84436
AN32A	4	0.42	1.6E-04	3.80883
ARP2	13	0.51	1.6E-04	3.79942
RBP56	2	0.39	1.6E-04	3.79507
AIMP1	3	0.24	1.7E-04	3.78068
KT3K	1	2.91	1.7E-04	3.76221
AIFM1	5	0.40	1.7E-04	3.75945
SRPRA	1	0.15	1.7E-04	3.75721
11-Sep	4	0.43	1.8E-04	3.744
GNPI1	4	0.39	1.8E-04	3.73779
MCTS1	1	0.43	1.9E-04	3.72862
SEM7A	8	5.49	1.9E-04	3.72538
RSMB, RSMN	3	0.45	2.1E-04	3.66776
CAPZB	15	0.49	2.2E-04	3.66314
LYAM1	3	11.5	2.3E-04	3.64608
AATC	8	0.57	2.3E-04	3.63283
WDR1	17	0.63	2.4E-04	3.61154
IF4H	1	8.60	2.5E-04	3.60206
PNOC	2	45.4	2.5E-04	3.59602
ACTN4	46	0.50	2.7E-04	3.57675
SRP14	3	0.42	2.7E-04	3.57528
HCFC1	2	0.37	2.7E-04	3.56527
RAB5C	2	0.40	2.7E-04	3.56511
CXCL9	8	147	2.8E-04	3.55705
AP2M1	2	0.39	2.9E-04	3.5397
KAD2	10	0.40	3.1E-04	3.50307
SF01	4	0.42	3.3E-04	3.47912
PTPRE	8	0.37	3.3E-04	3.47599
7-Sep	11	0.46	3.3E-04	3.47521
GRAB	2	18.1	3.4E-04	3.46916
HNRPD	9	0.45	3.4E-04	3.46332
NH2L1	1	0.42	3.7E-04	3.43192
PSB1	6	0.47	4.0E-04	3.3933
SPS1	3	0.38	4.1E-04	3.39073
PRDX2	8	0.56	4.1E-04	3.38977
TBL1X	1	0.25	4.1E-04	3.38595
CAZA2	5	0.50	4.1E-04	3.38426
GDIA	5	0.48	4.3E-04	3.36957
RAB2A	3	0.30	4.4E-04	3.35793
HMCS1	4	13.8	4.4E-04	3.35586
LANC1	4	0.42	4.6E-04	3.33913
HDHD2	2	0.41	4.8E-04	3.31912
ESYT1	1	0.29	4.8E-04	3.31677
DDAH2	5	0.37	5.0E-04	3.30417

COR1A	20	0.56	5.1E-04	3.29183
CATZ	4	3.33	5.3E-04	3.27548
DHX9	9	0.44	5.4E-04	3.26833
2-Sep	8	0.41	5.6E-04	3.25524
CAP1	24	0.56	5.6E-04	3.25065
VINC	35	0.51	5.6E-04	3.24841
COPE	5	0.49	5.8E-04	3.23897
RBMX	6	0.47	6.1E-04	3.21453
PARK7	11	0.47	6.2E-04	3.20859
RBP2	2	0.26	6.3E-04	3.19846
PGK1	24	0.57	6.4E-04	3.19716
ROAA	4	0.48	6.5E-04	3.18595
EIF3B	7	0.47	6.9E-04	3.15939
PSA1	11	0.52	7.0E-04	3.15633
SMRC2	4	0.25	7.0E-04	3.15627
SP16H	4	0.40	7.0E-04	3.15596
TRAD1	1	39.1	7.2E-04	3.14363
COPA	20	0.51	7.3E-04	3.13954
CELF2	1	0.41	7.4E-04	3.12819
SAHH	10	0.50	7.6E-04	3.1197
SF3B1	4	0.43	7.6E-04	3.11839
ENPP2	5	12.9	7.7E-04	3.11283
RL5	10	0.45	7.7E-04	3.11165
UBC9	2	0.31	7.8E-04	3.10807
APEX1	8	0.37	7.8E-04	3.10563
IF4A2	4	0.43	8.0E-04	3.0968
6PGL	8	0.45	8.3E-04	3.07956
ILF3	10	0.46	8.4E-04	3.07603
RMD3	1	0.26	8.5E-04	3.0689
PRDX6	13	0.51	8.8E-04	3.05567
AP1B1	9	0.51	9.0E-04	3.04793
ABHEB	3	0.43	9.1E-04	3.04206
ARP3	20	0.52	9.5E-04	3.02452
DDTL	2	0.33	9.9E-04	3.00542
TCRG1	1	0.36	1.0E-03	3
PML	1	0.35	1.0E-03	3
RAB1A	1	0.24	1.0E-03	3
KYNU	2	0.40	1.0E-03	3
CUL4B	3	0.46	1.0E-03	3
RHOA	4	0.51	1.0E-03	3
PSB3	4	0.47	1.0E-03	3
TSN	4	0.45	1.0E-03	3
PSA3	6	0.53	1.0E-03	3
MK01	6	0.52	1.0E-03	3

PSA5	6	0.52	1.0E-03	3
UBE2N	6	0.47	1.0E-03	3
SC23A	7	0.46	1.0E-03	3
SFPQ	9	0.65	1.0E-03	3
SNAA	9	0.53	1.0E-03	3
PSB4	9	0.49	1.0E-03	3
MDHC	11	0.53	1.0E-03	3
SLAF7	1	9.51	2.0E-03	2.69897
RUXGL	1	0.45	2.0E-03	2.69897
LSM3	1	0.44	2.0E-03	2.69897
DYL1	1	0.37	2.0E-03	2.69897
STRN3	1	0.25	2.0E-03	2.69897
LHPP	1	0.00	2.0E-03	2.69897
AIP	2	0.52	2.0E-03	2.69897
LZIC	2	0.40	2.0E-03	2.69897
XRN2	2	0.34	2.0E-03	2.69897
IL6	3	9.85	2.0E-03	2.69897
UCHL3	3	0.52	2.0E-03	2.69897
H2A1D	3	0.51	2.0E-03	2.69897
PSB10	3	0.50	2.0E-03	2.69897
NIF3L	3	0.43	2.0E-03	2.69897
SRP09	4	0.41	2.0E-03	2.69897
ISG15	5	24.5	2.0E-03	2.69897
MA1A1	5	4.49	2.0E-03	2.69897
FABP5	5	0.53	2.0E-03	2.69897
IAH1	5	0.37	2.0E-03	2.69897
CYC	5	0.36	2.0E-03	2.69897
PEBP1	5	0.32	2.0E-03	2.69897
PDC6I	6	0.57	2.0E-03	2.69897
CAN1	6	0.50	2.0E-03	2.69897
PNPH	6	0.47	2.0E-03	2.69897
HNRPL	7	0.54	2.0E-03	2.69897
DHX15	7	0.45	2.0E-03	2.69897
NPM	8	0.46	2.0E-03	2.69897
ARPC2	9	0.54	2.0E-03	2.69897
ILF2	9	0.48	2.0E-03	2.69897
SF3B3	10	0.54	2.0E-03	2.69897
NAGK	12	0.57	2.0E-03	2.69897
PSA6	12	0.54	2.0E-03	2.69897
CAZA1	12	0.52	2.0E-03	2.69897
ADA	15	0.41	2.0E-03	2.69897
CATA	15	0.41	2.0E-03	2.69897
GELS	23	0.78	2.0E-03	2.69897
GDIB	27	0.52	2.0E-03	2.69897

IQGA1	28	0.51	2.0E-03	2.69897
VIME	34	0.66	2.0E-03	2.69897
PP6R1	1	0.39	3.0E-03	2.52288
HS105	2	3.10	3.0E-03	2.52288
DNJA1	2	2.75	3.0E-03	2.52288
PLIN3	2	0.55	3.0E-03	2.52288
RBM8A	2	0.50	3.0E-03	2.52288
NUDT5	2	0.49	3.0E-03	2.52288
LSM8	2	0.45	3.0E-03	2.52288
SF3A1	2	0.42	3.0E-03	2.52288
UBC12	2	0.41	3.0E-03	2.52288
AP1S2	2	0.40	3.0E-03	2.52288
PP14B	2	0.36	3.0E-03	2.52288
NLTP	2	0.33	3.0E-03	2.52288
HSP13	3	4.27	3.0E-03	2.52288
AP2B1	3	0.44	3.0E-03	2.52288
DDX1	3	0.43	3.0E-03	2.52288
RS21	3	0.38	3.0E-03	2.52288
RL22	3	0.34	3.0E-03	2.52288
PSB9	4	0.53	3.0E-03	2.52288
CBX3	4	0.45	3.0E-03	2.52288
STX7	4	0.26	3.0E-03	2.52288
A16A1	5	0.47	3.0E-03	2.52288
F10A1	5	0.46	3.0E-03	2.52288
LAP2A	6	0.52	3.0E-03	2.52288
TPR	6	0.37	3.0E-03	2.52288
EF1G	10	0.62	3.0E-03	2.52288
COF1	10	0.60	3.0E-03	2.52288
DCPS	10	0.42	3.0E-03	2.52288
PSA7	11	0.52	3.0E-03	2.52288
EIFCL	11	0.51	3.0E-03	2.52288
TWF2	11	0.45	3.0E-03	2.52288
COPG1	12	0.55	3.0E-03	2.52288
STIP1	13	0.71	3.0E-03	2.52288
1433Z	14	0.55	3.0E-03	2.52288
TCPA	15	0.57	3.0E-03	2.52288
SQSTM	1	46.0	4.0E-03	2.39794
ASC	1	0.49	4.0E-03	2.39794
THOC4	1	0.45	4.0E-03	2.39794
H10	1	0.40	4.0E-03	2.39794
CDC73	1	0.36	4.0E-03	2.39794
LEO1	1	0.26	4.0E-03	2.39794
DDRGK	1	0.22	4.0E-03	2.39794
TSNAX	2	0.46	4.0E-03	2.39794

ERH	2	0.11	4.0E-03	2.39794
SYNC	3	0.61	4.0E-03	2.39794
PACN2	3	0.44	4.0E-03	2.39794
6-Sep	4	0.53	4.0E-03	2.39794
RO60	4	0.49	4.0E-03	2.39794
SCFD1	4	0.45	4.0E-03	2.39794
PA2G4	6	0.64	4.0E-03	2.39794
PSB2	6	0.52	4.0E-03	2.39794
ARPC3	7	0.59	4.0E-03	2.39794
EF1D	7	0.58	4.0E-03	2.39794
RS12	7	0.55	4.0E-03	2.39794
ARPC4	7	0.52	4.0E-03	2.39794
9-Sep	8	0.35	4.0E-03	2.39794
ESTD	9	0.57	4.0E-03	2.39794
PSB8	9	0.52	4.0E-03	2.39794
LYSC	9	0.48	4.0E-03	2.39794
GLOD4	9	0.45	4.0E-03	2.39794
H4	12	0.63	4.0E-03	2.39794
EIF3A	13	0.49	4.0E-03	2.39794
TAGL2	13	0.48	4.0E-03	2.39794
PSA4	14	0.49	4.0E-03	2.39794
PLEC	34	0.52	4.0E-03	2.39794
VAS1	1	10.6	0.01	2.30103
LIMD2	1	2.99	0.01	2.30103
LCK	1	0.40	0.01	2.30103
DKC1	1	0.36	0.01	2.30103
SRS11	1	0.12	0.01	2.30103
DCNL1	2	0.37	5.0E-03	2.30103
HNRDL	3	0.64	5.0E-03	2.30103
DEK	3	0.52	5.0E-03	2.30103
EIF3E	3	0.50	5.0E-03	2.30103
CPNS1	4	0.56	5.0E-03	2.30103
VTDB	6	1.28	5.0E-03	2.30103
DX39B	7	0.54	5.0E-03	2.30103
RL12	7	0.48	5.0E-03	2.30103
RAN	8	0.54	5.0E-03	2.30103
TCPH	13	0.56	5.0E-03	2.30103
TCPG	14	0.58	5.0E-03	2.30103
TCPD	16	0.56	5.0E-03	2.30103
TCPB	17	0.58	5.0E-03	2.30103
UBA1	24	0.58	5.0E-03	2.30103
TIA1	1	0.61	0.01	2.22185
CPSF6	1	0.51	0.01	2.22185
PSMD5	1	0.45	0.01	2.22185

ANM1	2	0.49	6.0E-03	2.22185
SMU1	2	0.46	6.0E-03	2.22185
PUF60	2	0.46	6.0E-03	2.22185
NGLY1	2	0.30	6.0E-03	2.22185
HCLS1	3	2.94	6.0E-03	2.22185
RPE	3	0.48	6.0E-03	2.22185
GSHB	4	0.54	6.0E-03	2.22185
HMGB2	6	0.52	6.0E-03	2.22185
ACPH	6	0.51	6.0E-03	2.22185
UB2V1	7	0.49	6.0E-03	2.22185
GSTP1	9	0.53	6.0E-03	2.22185
MVP	12	0.65	6.0E-03	2.22185
1433E	13	0.54	6.0E-03	2.22185
HLAE	1	29.6	0.01	2.1549
NTF2	1	0.43	0.01	2.1549
PFD5	1	0.36	0.01	2.1549
PP14A	1	0.32	0.01	2.1549
CSTF3	1	0.26	0.01	2.1549
EIF3I	5	0.53	7.0E-03	2.1549
SAE2	6	0.53	7.0E-03	2.1549
PSA2	7	0.45	7.0E-03	2.1549
RSSA	8	0.55	7.0E-03	2.1549
BUB3	8	0.47	7.0E-03	2.1549
COPD	8	0.47	7.0E-03	2.1549
HNRPC	12	0.58	7.0E-03	2.1549
PROF1	13	0.60	7.0E-03	2.1549
1433B	13	0.59	7.0E-03	2.1549
ENOA	20	0.54	7.0E-03	2.1549
DDX17	1	0.44	0.01	2.09691
WDR82	1	0.40	0.01	2.09691
SSRP1	2	0.45	8.0E-03	2.09691
OSGEP	2	0.43	8.0E-03	2.09691
CDC37	5	0.48	8.0E-03	2.09691
SYEP	6	0.39	8.0E-03	2.09691
AN32B	7	0.48	8.0E-03	2.09691
VATA	9	0.63	8.0E-03	2.09691
PSME1	9	0.57	8.0E-03	2.09691
PSA	12	0.53	8.0E-03	2.09691
2AAA	14	0.54	8.0E-03	2.09691
CLH1	37	0.61	8.0E-03	2.09691
IRF4	1	5.72	0.01	2.04576
B2MG	1	4.21	0.01	2.04576
STX12	1	0.45	0.01	2.04576
PLCB3	1	0.39	0.01	2.04576

DEOC	1	0.36	0.01	2.04576
REPI1	1	0.18	0.01	2.04576
P66A	1	0.18	0.01	2.04576
VP26A	2	0.60	9.0E-03	2.04576
CPSF5	2	0.45	9.0E-03	2.04576
UB2L3	6	0.61	9.0E-03	2.04576
HDGF	7	0.58	9.0E-03	2.04576
TCPZ	8	0.60	9.0E-03	2.04576
PPIA	14	0.60	9.0E-03	2.04576
LMNB1	21	0.55	9.0E-03	2.04576
GLYG	1	0.48	0.01	2
LSM5	1	0.44	0.01	2
SART3	1	0.37	0.01	2
SAP	3	2.68	0.01	2
UFM1	3	0.48	0.01	2
XPP1	4	0.56	0.01	2
EWS	4	0.52	0.01	2
EIF3D	5	0.59	0.01	2
FUBP1	6	0.58	0.01	2
RBBP4	9	0.49	0.01	2
PLSL	45	0.57	0.01	2
MYH9	78	0.63	0.01	2
SMD1	1	0.36	0.01	1.95861
NOLC1	1	0.29	0.01	1.95861
SEC13	2	0.55	0.01	1.95861
CZIB	2	0.48	0.01	1.95861
PFD2	2	0.46	0.01	1.95861
LYPA1	3	0.53	0.01	1.95861
API5	3	0.46	0.01	1.95861
ARF1, ARF3	4	0.39	0.01	1.95861
RS28	5	0.58	0.01	1.95861
COPB	7	0.52	0.01	1.95861
TPIS	15	0.61	0.01	1.95861
IRF8	1	83.8	0.01	1.92082
THMS2	1	14.2	0.01	1.92082
COPZ1	1	0.51	0.01	1.92082
BAP18	1	0.49	0.01	1.92082
LRC59	1	0.45	0.01	1.92082
BIN1	1	0.45	0.01	1.92082
GLGB	2	0.50	0.01	1.92082
K1C17	2	0.21	0.01	1.92082
GMFG	3	0.57	0.01	1.92082
PLPP	3	0.30	0.01	1.92082
DBNL	9	0.69	0.01	1.92082

**TPM4**	**11**	**0.60**	**0.01**	**1.92082**
**IPO5**	**12**	**0.49**	**0.01**	**1.92082**
**LDHB**	**13**	**0.56**	**0.01**	**1.92082**
**KCAB2**	**1**	**0.44**	**0.01**	**1.88606**
**EIF3K**	**2**	**0.51**	**0.01**	**1.88606**
**IF1AX**	**3**	**0.52**	**0.01**	**1.88606**
**SPB8**	**4**	**0.49**	**0.01**	**1.88606**
**HLAA**	**5**	**2.71**	**0.01**	**1.88606**
**GPX1**	**5**	**0.55**	**0.01**	**1.88606**
**XPO1**	**7**	**0.59**	**0.01**	**1.88606**
**KCD12**	**8**	**0.67**	**0.01**	**1.88606**
**DDB1**	**9**	**0.50**	**0.01**	**1.88606**
**ACTS**	**15**	**0.77**	**0.01**	**1.88606**
**TCPQ**	**18**	**0.63**	**0.01**	**1.88606**
**RN213**	**1**	**17.9**	**0.01**	**1.85387**
**NC2A**	**1**	**0.47**	**0.01**	**1.85387**
**RFA3**	**1**	**0.44**	**0.01**	**1.85387**
**GMPPB**	**1**	**0.34**	**0.01**	**1.85387**
**FCL**	**2**	**0.56**	**0.01**	**1.85387**
**THOP1**	**2**	**0.49**	**0.01**	**1.85387**
**ABRAL**	**3**	**0.50**	**0.01**	**1.85387**
**SMD3**	**4**	**0.51**	**0.01**	**1.85387**
**1433F**	**6**	**0.56**	**0.01**	**1.85387**
**PARP1**	**8**	**0.41**	**0.01**	**1.85387**
**IFIT1**	**1**	**8.42**	**0.02**	**1.82391**
**MTNB**	**1**	**0.35**	**0.02**	**1.82391**
**ELAV1**	**3**	**0.49**	**0.02**	**1.82391**
**GSHR**	**4**	**0.59**	**0.02**	**1.82391**
**FUBP2**	**6**	**0.63**	**0.02**	**1.82391**
**6PGD**	**12**	**0.63**	**0.02**	**1.82391**
**COPB2**	**14**	**0.57**	**0.02**	**1.82391**
**TERA**	**30**	**0.59**	**0.02**	**1.82391**
**CMPK2**	**1**	**29.2**	**0.02**	**1.79588**
**SWP70**	**1**	**2.76**	**0.02**	**1.79588**
**APOA1**	**1**	**0.69**	**0.02**	**1.79588**
**CNPY3**	**1**	**0.58**	**0.02**	**1.79588**
**BROX**	**1**	**0.47**	**0.02**	**1.79588**
**LRC47**	**1**	**0.34**	**0.02**	**1.79588**
**CATS**	**2**	**6.80**	**0.02**	**1.79588**
**GMPR2**	**2**	**0.54**	**0.02**	**1.79588**
**IF4E**	**2**	**0.53**	**0.02**	**1.79588**
**MGN2**	**2**	**0.38**	**0.02**	**1.79588**
**AP1M1**	**3**	**0.39**	**0.02**	**1.79588**
**HNRPR**	**5**	**0.68**	**0.02**	**1.79588**

SET	6	0.53	0.02	1.79588
PGM2	8	0.50	0.02	1.79588
HNRPU	10	0.54	0.02	1.79588
PRDX1	12	0.64	0.02	1.79588
ARC1B	15	0.67	0.02	1.79588
TRFE	65	0.75	0.02	1.79588
DDX3X	1	2.31	0.02	1.76955
SFT2B	1	0.43	0.02	1.76955
PLRG1	1	0.37	0.02	1.76955
SMCA4	1	0.29	0.02	1.76955
CNN2	4	0.54	0.02	1.76955
IF2G	5	0.58	0.02	1.76955
PTBP1	6	0.48	0.02	1.76955
BUD31	1	0.50	0.02	1.74473
LSM4	1	0.46	0.02	1.74473
AP1G1	2	0.49	0.02	1.74473
CHM1B	2	0.45	0.02	1.74473
LIS1	4	0.57	0.02	1.74473
GDS1	5	0.52	0.02	1.74473
RFA1	6	0.55	0.02	1.74473
HSP7C	28	0.63	0.02	1.74473
PIPNB	2	0.62	0.02	1.72125
FKB15	3	0.58	0.02	1.72125
IF4A3	3	0.55	0.02	1.72125
1433T	5	0.59	0.02	1.72125
SIAS	5	0.46	0.02	1.72125
MARE1	6	0.49	0.02	1.72125
MYL6	7	0.66	0.02	1.72125
H2B2F	10	0.60	0.02	1.72125
TMOD3	1	0.53	0.02	1.69897
HEBP1	1	0.49	0.02	1.69897
2ABA	2	0.61	0.02	1.69897
SH3L3	3	0.55	0.02	1.69897
EIF3H	4	0.60	0.02	1.69897
NDKB	9	0.55	0.02	1.69897
DPP3	14	0.59	0.02	1.69897
PDC10	1	0.48	0.02	1.67778
MTA2	2	0.39	0.02	1.67778
SMC3	2	0.28	0.02	1.67778
KINH	5	0.66	0.02	1.67778
MGN	1	0.49	0.02	1.65758
CSTN1	2	5.06	0.02	1.65758
SF3B6	2	0.47	0.02	1.65758
PPM1G	4	0.46	0.02	1.65758

YTDC1	1	0.40	0.02	1.63827
CSN4	2	0.54	0.02	1.63827
UGPA	3	0.52	0.02	1.63827
ADK	3	0.52	0.02	1.63827
DEST	3	0.50	0.02	1.63827
EMAL2	4	0.60	0.02	1.63827
SYK	6	0.61	0.02	1.63827
MAT2B	6	0.47	0.02	1.63827
PSME2	10	0.60	0.02	1.63827
RHG01	1	0.81	0.02	1.61979
DHSO	2	0.47	0.02	1.61979
DHPR	4	0.41	0.02	1.61979
GBP3	1	21.3	0.03	1.60206
MATR3	1	0.53	0.03	1.60206
EF1D	1	0.49	0.03	1.60206
PLST	1	0.41	0.03	1.60206
DP13A	2	0.42	0.03	1.60206
H33	3	0.60	0.03	1.60206
MIF	3	0.51	0.03	1.60206
TCPE	9	0.61	0.03	1.60206
CAND1	10	0.52	0.03	1.60206
SYVC	1	0.49	0.03	1.58503
H13	2	0.31	0.03	1.58503
SND1	9	0.68	0.03	1.58503
SRP72	1	5.71	0.03	1.56864
VATE1	1	0.56	0.03	1.56864
RL7	2	0.57	0.03	1.56864
GSLG1	3	3.18	0.03	1.56864
F16P1	6	0.57	0.03	1.56864
TRYP_PIG	10	1.69	0.03	1.56864
ALDOA	22	0.66	0.03	1.56864
CSN5	1	0.57	0.03	1.55284
DNJC9	1	0.49	0.03	1.55284
RUXF	2	0.50	0.03	1.55284
NP1L4	5	0.48	0.03	1.55284
AK1A1	7	0.60	0.03	1.55284
PTN6	16	0.64	0.03	1.55284
TALDO	16	0.59	0.03	1.55284
TPM2	1	0.45	0.03	1.5376
PTPA	5	0.58	0.03	1.5376
ACTB	21	0.70	0.03	1.5376
UBL4A	1	0.37	0.03	1.52288
MTAP	1	0.36	0.03	1.52288
AP1B1	1	0.16	0.03	1.52288

GBRL2	2	0.57	0.03	1.52288
LZTL1	2	0.43	0.03	1.52288
CD2AP	5	0.58	0.03	1.52288
RACK1	8	0.63	0.03	1.52288
NPC2	1	2.59	0.03	1.50864
CRYM	2	0.26	0.03	1.50864
GDIR1	7	0.61	0.03	1.50864
RLA0	9	0.57	0.03	1.49485
TPM3	20	0.65	0.03	1.49485
STAT3	1	6.31	0.03	1.48149
LXN	1	0.45	0.03	1.48149
TMA7	2	0.57	0.03	1.48149
EIF3G	2	0.53	0.03	1.48149
IMPA1	7	0.50	0.03	1.48149
LKHA4	18	0.61	0.03	1.48149
TXD17	2	0.58	0.03	1.46852
GFRP	2	0.26	0.03	1.46852
SPF27	1	0.53	0.04	1.45593
SUMO3	1	0.51	0.04	1.45593
SC31A	2	0.57	0.04	1.45593
TSP1	1	0.74	0.04	1.4437
H2A1B	1	0.58	0.04	1.4437
OAS3	2	2.91	0.04	1.4437
PRDX5	3	0.56	0.04	1.4437
PSB6	3	0.51	0.04	1.4437
OTUB1	4	0.55	0.04	1.4437
DNJC8	7	0.53	0.04	1.4437
K2C6B	9	0.35	0.04	1.4437
RCC2	13	0.57	0.04	1.4437
PHF5A	1	0.26	0.04	1.4318
CDC42	4	0.65	0.04	1.4318
SYSC	5	0.65	0.04	1.4318
PGM1	9	0.54	0.04	1.42022
ARC1A	2	0.55	0.04	1.40894
DAZP1	2	0.37	0.04	1.40894
HLAB	4	2.56	0.04	1.40894
IDHC	9	0.64	0.04	1.40894
CADH1	1	3.22	0.04	1.39794
DENR	1	0.56	0.04	1.39794
RGS10	1	0.56	0.04	1.39794
SASH3	1	0.54	0.04	1.39794
HNRPQ	4	0.65	0.04	1.39794
ARK72	1	0.41	0.04	1.38722
SPB6	2	0.55	0.04	1.38722

**ECHD1**	**2**	**0.49**	**0.04**	**1.38722**
**SMC1A**	**3**	**0.50**	**0.04**	**1.38722**
**CBX5**	**3**	**0.46**	**0.04**	**1.38722**
**LPXN**	**1**	**0.62**	**0.04**	**1.37675**
**EIF3F**	**2**	**0.66**	**0.04**	**1.37675**
**DDX5**	**2**	**0.60**	**0.04**	**1.37675**
**ML12A**	**5**	**0.75**	**0.04**	**1.37675**
**PP1A**	**7**	**0.63**	**0.04**	**1.37675**
**UFC1**	**3**	**0.53**	**0.04**	**1.35655**
**TPM3**	**1**	**0.67**	**0.05**	**1.34679**
**FMC1**	**1**	**0.64**	**0.05**	**1.34679**
**AP3D1**	**2**	**0.17**	**0.05**	**1.34679**
**PTMA**	**4**	**3.23**	**0.05**	**1.34679**
**IMA3**	**1**	**2.33**	**0.05**	**1.33724**
**RBBP7**	**1**	**0.55**	**0.05**	**1.33724**
**VAMP7**	**1**	**0.41**	**0.05**	**1.33724**
**STRAP**	**3**	**0.58**	**0.05**	**1.33724**
**RAB8A**	**3**	**0.57**	**0.05**	**1.33724**
**FKB1A**	**3**	**0.41**	**0.05**	**1.33724**
**IF16**	**5**	**0.56**	**0.05**	**1.33724**
**HSP74**	**12**	**0.62**	**0.05**	**1.33724**
**TFIP8**	**1**	**0.64**	**0.05**	**1.3279**
**MOB1A**	**1**	**0.40**	**0.05**	**1.3279**
**PEBB**	**1**	**0.18**	**0.05**	**1.3279**
**GLRX3**	**1**	**3.93**	**0.05**	**1.31876**
**PRPS2**	**3**	**0.56**	**0.05**	**1.31876**
**G3BP1**	**5**	**0.67**	**0.05**	**1.31876**
**GSTO1**	**6**	**0.57**	**0.05**	**1.31876**
**GYS1**	**1**	**3.86**	**0.05**	**1.3098**
**CSTF2**	**1**	**0.51**	**0.05**	**1.3098**
**LC7L3**	**1**	**0.50**	**0.05**	**1.3098**
**PP2AA**	**3**	**0.54**	**0.05**	**1.3098**
**CSN2**	**1**	**0.49**	**0.05**	**1.30103**
**PURA**	**2**	**0.58**	**0.05**	**1.30103**
**ACLY**	**14**	**0.66**	**0.05**	**1.30103**
**HNRPK**	**15**	**0.71**	**0.05**	**1.30103**
**CY24B**	**1**	**0.58**	**0.05**	**1.29243**
**RBM12**	**1**	**0.45**	**0.05**	**1.29243**
**SMD2**	**2**	**0.50**	**0.05**	**1.29243**
**H2A2B**	**4**	**0.56**	**0.05**	**1.29243**
**BCLF1**	**2**	**0.55**	**0.05**	**1.284**
**PAIP1**	**3**	**0.54**	**0.05**	**1.284**
**SRSF6**	**2**	**3.15**	**0.05**	**1.27572**
**EIF3L**	**6**	**0.59**	**0.05**	**1.27572**

MX1	15	2.46	0.05	1.27572
FUS	1	2.11	0.05	1.26761
SYCC	1	0.56	0.05	1.26761
DCTD	1	0.43	0.05	1.26761
ARPC5	4	0.51	0.05	1.26761
GRB2	7	0.66	0.05	1.26761
CFAH	1	1.55	0.06	1.25964
HMCN1	1	1.32	0.06	1.25964
PP1B	1	0.62	0.06	1.25964
DC1I2	1	0.27	0.06	1.25964
NOP56	2	0.55	0.06	1.25964
TSTD1	2	0.51	0.06	1.25964
IST1	2	0.57	0.06	1.25181
1433B	1	0.62	0.06	1.24413
LYRIC	1	0.11	0.06	1.24413
NUCKS	2	0.40	0.06	1.24413
ARHL2	3	0.52	0.06	1.23657
GMPPA	1	0.51	0.06	1.22915
EXOS4	1	0.24	0.06	1.22915
THOC2	1	0.35	0.06	1.22185
IF2P	2	0.37	0.06	1.22185
VPS35	4	0.64	0.06	1.22185
CFAB	1	7.00	0.06	1.20761
SRGN	1	116	0.06	1.20066
DCUP	1	0.27	0.06	1.20066
OLA1	2	0.55	0.06	1.20066
GBB1	9	0.67	0.06	1.20066
HARS1	11	0.59	0.06	1.20066
XRCC5	14	0.67	0.06	1.20066
SYWC	15	1.76	0.06	1.20066
HDGR2	1	0.10	0.06	1.19382
CPPED	2	0.52	0.06	1.19382
RRBP1	21	0.62	0.06	1.19382
PYGB	1	0.62	0.07	1.18709
SYDC	5	0.71	0.07	1.18709
UBL5	1	1.86	0.07	1.18046
K1C16	11	0.23	0.07	1.18046
LA	9	0.63	0.07	1.17393
UB2V2	1	0.48	0.07	1.1549
GGCT	3	0.49	0.07	1.1549
NUCL	17	0.61	0.07	1.1549
DCD	1	1.47	0.07	1.14874
PFD3	2	0.52	0.07	1.14874
OSBP1	1	0.51	0.07	1.14267

**ERF1**	**2**	**0.55**	**0.07**	**1.14267**
**NP1L1**	**5**	**0.58**	**0.07**	**1.14267**
**EPS15**	**1**	**0.44**	**0.07**	**1.13668**
**RTRAF**	**2**	**0.61**	**0.07**	**1.13077**
**SRSF1**	**7**	**0.65**	**0.07**	**1.13077**
**EIF3J**	**1**	**0.53**	**0.08**	**1.12494**
**ACTG**	**2**	**0.39**	**0.08**	**1.12494**
**CBR1**	**3**	**0.34**	**0.08**	**1.12494**
**XRCC6**	**12**	**0.66**	**0.08**	**1.12494**
**PUR6**	**5**	**0.62**	**0.08**	**1.11919**
**TRXR1**	**7**	**0.61**	**0.08**	**1.11919**
**IF4G2**	**1**	**23.0**	**0.08**	**1.10791**
**3BP1**	**2**	**2.05**	**0.08**	**1.10791**
**TBCA**	**4**	**0.68**	**0.08**	**1.10791**
**IF4G1**	**1**	**4.24**	**0.08**	**1.10237**
**CCAR2**	**1**	**0.60**	**0.08**	**1.10237**
**U2AF2**	**2**	**0.53**	**0.08**	**1.09691**
**TIMP1**	**3**	**0.64**	**0.08**	**1.09691**
**FLII**	**1**	**0.75**	**0.08**	**1.09151**
**ELOC**	**1**	**0.58**	**0.08**	**1.09151**
**LDHA**	**16**	**0.61**	**0.08**	**1.09151**
**RMD1**	**1**	**0.43**	**0.08**	**1.08619**
**MAP1A**	**1**	**0.35**	**0.08**	**1.08619**
**NECP2**	**1**	**0.67**	**0.08**	**1.08092**
**1433G**	**6**	**0.62**	**0.08**	**1.08092**
**PTMS**	**2**	**0.43**	**0.08**	**1.07572**
**ANR44**	**1**	**0.65**	**0.09**	**1.07058**
**G3P**	**18**	**0.68**	**0.09**	**1.0655**
**ERP29**	**6**	**0.73**	**0.09**	**1.05552**
**TKT**	**29**	**0.65**	**0.09**	**1.05552**
**STK24**	**1**	**0.54**	**0.09**	**1.05061**
**HAT1**	**1**	**0.38**	**0.09**	**1.05061**
**THIC**	**2**	**0.58**	**0.09**	**1.05061**
**PGAM1**	**16**	**0.65**	**0.09**	**1.05061**
**FNTA**	**1**	**0.55**	**0.09**	**1.04576**
**B4GT1**	**2**	**2.19**	**0.09**	**1.04576**
**ENOPH**	**2**	**0.58**	**0.09**	**1.04576**
**TCP4**	**8**	**0.58**	**0.09**	**1.04096**
**TOP1**	**1**	**0.46**	**0.09**	**1.03621**
**DNJA2**	**2**	**1.59**	**0.09**	**1.03621**
**AMPB**	**8**	**0.58**	**0.09**	**1.03152**
**PUR8**	**1**	**0.54**	**0.09**	**1.02687**
**PLEK**	**7**	**1.90**	**0.09**	**1.02687**
**ILEU**	**13**	**0.72**	**0.09**	**1.02687**

TRY1	1	1.49	0.10	1.02228
CK054	1	0.41	0.10	1.01773
DNM1L	1	0.71	0.10	1.01323
DDX23	1	0.19	0.10	1.01323
PDIA3	13	0.72	0.10	1.01323
SF3B2	4	0.62	0.10	1.00877
NDRG1	2	0.39	0.10	1.00436
HLAC	3	3.17	0.10	1
H31	2	0.65	0.10	0.99568
CUTA	3	0.62	0.10	0.9914
U5S1	1	0.60	0.10	0.98716
NRDC	1	0.49	0.10	0.98716
PIMT	4	0.38	0.10	0.98716
PLPHP	2	0.63	0.10	0.98297
TEBP	3	1.84	0.10	0.98297
ALDOC	3	0.65	0.10	0.98297
EF2	32	0.73	0.10	0.98297
E2AK2	1	65.3	0.11	0.97469
RS30	1	2.38	0.11	0.97469
UBP14	4	0.69	0.11	0.97469
MYDGF	2	0.44	0.11	0.96257
FKBP4	5	0.63	0.11	0.96257
GDIR2	10	0.70	0.11	0.96257
B2CL2	1	0.30	0.11	0.95861
K2C6C	1	0.42	0.11	0.95468
FUMH	2	0.66	0.11	0.95078
DUT	1	2.73	0.12	0.9393
SPTN1	4	0.61	0.12	0.9393
IF2A	3	0.65	0.12	0.93554
AP2A1	5	0.61	0.12	0.93554
WDR48	1	0.25	0.12	0.93181
BPNT1	2	0.66	0.12	0.92812
TBA1A	1	1.82	0.12	0.92445
ULA1	1	0.52	0.12	0.92445
INS	2	1.16	0.12	0.92445
IL16	2	0.62	0.12	0.92445
GLRX1	2	0.57	0.12	0.92445
HPRT	3	0.62	0.12	0.92445
GUAA	1	0.44	0.12	0.91009
SCRN1	3	1.55	0.12	0.91009
HORN	4	0.43	0.12	0.91009
MESD	1	0.68	0.12	0.90658
PTGDS	3	3.78	0.12	0.90658
TSSK4	1	1.25	0.13	0.90309

ELMO1	2	0.74	0.13	0.90309
FHL1	3	0.22	0.13	0.90309
ABI1	1	0.59	0.13	0.89963
CAB39	1	0.51	0.13	0.8962
SH3L1	2	0.62	0.13	0.8962
EDF1	3	0.64	0.13	0.8962
LSP1	8	0.64	0.13	0.8962
EIF1	1	1.51	0.13	0.89279
5NTC	1	0.52	0.13	0.89279
CCL19	1	1,000	0.13	0.88941
PTPRS	5	1.54	0.13	0.88273
CIRBP	1	0.57	0.13	0.87943
CYFP2	3	0.68	0.13	0.87943
DCTN1	3	0.55	0.13	0.87943
UBP5	3	0.52	0.13	0.87615
LEG1	8	0.69	0.13	0.87615
PABP1	10	0.71	0.13	0.8729
FKBP3	2	0.64	0.14	0.86967
K1C14	5	0.19	0.14	0.86967
PUR2	6	1.69	0.14	0.86967
PPCE	2	0.66	0.14	0.85699
QOR	4	0.46	0.14	0.85699
NMT1	3	0.66	0.14	0.85387
K2C5	14	0.29	0.14	0.85387
AP3S1	1	0.59	0.14	0.84466
ALDR	5	0.60	0.14	0.84466
RBM14	1	1.79	0.15	0.83565
PSPC1	1	0.63	0.15	0.83565
CHM4B	2	1.48	0.15	0.83565
TSYL2	1	2.14	0.15	0.83268
NACAM	2	0.69	0.15	0.83268
RS20	2	0.70	0.15	0.82974
DDX6	4	0.72	0.15	0.82974
TCEA1	4	0.45	0.15	0.82974
KSYK	1	1.48	0.15	0.82681
PCBP2	6	0.76	0.15	0.82681
IF6	4	0.81	0.15	0.82391
HMGB1	6	0.59	0.15	0.82102
EXOS9	1	0.64	0.15	0.81816
MICA1	1	0.60	0.15	0.81816
DCXR	2	0.67	0.16	0.80967
SPTB2	8	0.61	0.16	0.80967
G6PI	15	0.71	0.16	0.80967
YBOX1	7	0.66	0.16	0.80688

IN35	1	1.32	0.16	0.80134
AP1G2	2	0.31	0.16	0.7986
BTF3	1	0.66	0.16	0.79588
4F2	1	0.55	0.16	0.79588
K2C78	3	0.25	0.16	0.79588
RPR1B	1	0.60	0.16	0.79048
RS8	7	0.66	0.16	0.79048
ANXA5	17	0.78	0.16	0.79048
ROA2	19	0.76	0.16	0.78781
PPARD	1	1.18	0.16	0.78516
C19L1	1	0.43	0.16	0.78516
MP2K2	1	1.60	0.17	0.78252
HGFA	1	1.65	0.17	0.77989
MFAP1	1	0.51	0.17	0.77728
SUMO1	1	0.45	0.17	0.77728
S10AA	2	0.69	0.17	0.77211
IFNG	1	24.2	0.17	0.76955
AGFG1	2	0.60	0.17	0.76955
RAB8B	1	2.12	0.17	0.76195
ACL6A	1	0.64	0.17	0.75945
ISOC1	2	0.55	0.17	0.75945
WASP	4	0.61	0.17	0.75945
HV373	1	4.68	0.18	0.75696
DESP	3	0.16	0.18	0.75696
HS90A	33	0.69	0.18	0.75696
RS27A	2	0.64	0.18	0.74232
ROA1	12	0.74	0.18	0.73993
EIF2A	1	0.41	0.18	0.73518
RS16	4	0.61	0.18	0.73518
THUM1	1	0.63	0.19	0.73283
TIGAR	1	0.61	0.19	0.73283
UB2J1	1	0.11	0.19	0.73283
PPM1F	2	0.65	0.19	0.72584
H32	2	0.73	0.19	0.7167
PTN11	2	0.63	0.19	0.71444
RS11	2	0.64	0.20	0.70997
PCBP1	11	0.80	0.20	0.70997
RL32	1	0.56	0.20	0.70553
HGS	1	0.67	0.20	0.70333
RL17	1	0.65	0.20	0.69897
TRA2B	2	0.75	0.20	0.69897
GMIP	1	0.52	0.20	0.6968
RL23A	3	0.65	0.20	0.69465
HEBP2	1	0.64	0.20	0.6925

ABRX2	3	0.67	0.20	0.6925
K1C9	35	0.48	0.20	0.69037
K1C10	27	0.30	0.21	0.68613
PLCG2	1	0.65	0.21	0.68403
RU2A	1	0.51	0.21	0.67985
DSC1	1	0.23	0.21	0.67985
SARNP	2	0.68	0.21	0.67778
ADHX	5	0.64	0.21	0.67778
TPD54	1	0.69	0.21	0.67572
NIT2	1	0.49	0.21	0.67366
RL18	2	0.67	0.21	0.67366
GIT2	2	0.50	0.21	0.67162
CATG	1	2.80	0.21	0.66959
ZRAB2	1	0.60	0.22	0.66756
RS10	1	0.78	0.22	0.65758
HS71A	21	1.21	0.22	0.65758
K22E	20	0.31	0.22	0.65561
CYTC	3	0.75	0.22	0.65365
AHNK	5	0.74	0.22	0.65365
K2C1	38	0.37	0.22	0.65365
PSDE	1	0.77	0.22	0.64975
H2B1B	2	0.70	0.23	0.64589
SGTA	1	0.54	0.23	0.64016
BST2	2	2.46	0.23	0.63827
TBB6	4	0.61	0.23	0.63451
CCD50	2	0.60	0.23	0.63264
TYB4	1	0.78	0.24	0.62893
CIAO1	1	0.60	0.24	0.62893
EIF3M	2	0.67	0.24	0.62893
PFKAL	2	0.80	0.24	0.62709
BZW1	1	1.62	0.24	0.62525
DYH8	1	1.12	0.24	0.62525
BLMH	1	0.72	0.24	0.6216
RL36	1	0.60	0.24	0.6216
MTND	1	0.38	0.24	0.61798
TPP2	4	0.72	0.24	0.61618
LMNA	5	0.63	0.24	0.61618
HEM2	1	0.52	0.24	0.61439
DIAP1	4	0.78	0.24	0.61439
TPD52	1	0.44	0.24	0.61261
RS3A	9	0.72	0.24	0.61261
RL27	2	0.72	0.25	0.60906
MYPT1	1	0.59	0.25	0.60555
RS3	6	0.72	0.25	0.60555

FBX6	1	2.89	0.25	0.60033
SGT1	2	0.71	0.25	0.59688
AMY1	2	0.77	0.26	0.59346
PSMD9	3	0.75	0.26	0.59176
PA1B2	1	0.77	0.26	0.59007
LTOR1	1	0.48	0.26	0.59007
NIBA1	1	2.57	0.26	0.5867
RAGP1	2	0.65	0.26	0.5867
SORCN	1	0.73	0.26	0.58503
PTGR1	2	0.37	0.26	0.58503
TXNL1	2	0.76	0.26	0.5817
SAMH1	19	0.85	0.27	0.57675
USP9X	1	0.55	0.27	0.57349
OSTF1	2	0.79	0.27	0.56543
AL9A1	1	0.63	0.27	0.56225
RL4	5	0.72	0.28	0.55909
HV64D	1	0.72	0.28	0.55596
RPAB3	1	0.37	0.28	0.5544
PAIRB	2	0.79	0.28	0.5544
RL30	2	0.70	0.28	0.5544
PDIA1	12	0.72	0.28	0.55129
EZRI	17	0.73	0.28	0.54975
DYHC1	1	0.76	0.28	0.54668
PRS8	1	1.43	0.29	0.5391
RL11	3	1.38	0.29	0.5391
CSK2B	1	0.76	0.29	0.5376
HMGN2	2	0.65	0.30	0.52578
NONO	3	0.80	0.30	0.52578
ANXA6	6	0.78	0.30	0.52578
SYFA	1	0.71	0.30	0.52288
RL21	1	0.69	0.30	0.52288
RL10A	5	0.78	0.30	0.52288
PSME3	2	0.71	0.30	0.52143
LV469	1	2.47	0.30	0.51999
PHP14	1	0.37	0.30	0.51999
CNTN1	2	1.08	0.30	0.51856
U2AF5	1	0.74	0.30	0.51713
SKP1	1	0.48	0.31	0.5157
A2MG	1	1.41	0.31	0.51286
RS7	2	0.67	0.31	0.51145
OXSR1	1	0.72	0.31	0.50864
RL35A	1	2.00	0.31	0.50724
HYOU1	3	1.34	0.31	0.50724
BGH3	7	0.81	0.31	0.50585

CO4A	2	1.16	0.31	0.50446
ABCE1	1	0.61	0.31	0.50307
PIN1	1	0.66	0.32	0.50169
HNRH1	9	0.84	0.32	0.50169
SPB12	1	0.40	0.32	0.50031
LRRF1	1	0.86	0.32	0.49894
GILT	1	1.34	0.32	0.49757
RAB10	1	0.79	0.32	0.48945
NPL4	1	0.75	0.32	0.48945
DYN2	3	0.70	0.32	0.48945
RL3	6	0.75	0.32	0.48945
PDCD6	1	0.72	0.33	0.48812
BLVRB	1	1.71	0.33	0.48413
RS4X	2	0.67	0.33	0.48413
HS90B	17	0.79	0.33	0.4828
CH60	3	1.63	0.33	0.48149
SAFB1	2	0.67	0.33	0.48017
URP2	11	0.81	0.33	0.48017
FRIL	1	1.88	0.33	0.47886
AIF1	2	0.81	0.33	0.47625
SAP18	1	0.63	0.34	0.47237
PSIP1	1	0.50	0.34	0.47237
S10AB	1	0.75	0.34	0.46852
GRHPR	1	0.68	0.34	0.46725
UBR4	1	2.28	0.34	0.46597
GLU2B	1	0.74	0.34	0.46597
NUDC	1	0.74	0.34	0.46471
NT5D1	1	0.67	0.34	0.46471
USO1	7	0.78	0.34	0.46471
CAR11	1	0.46	0.35	0.46218
IPP2B	1	1.98	0.35	0.46092
HSPB1	1	0.64	0.35	0.45842
PSD11	3	1.31	0.35	0.45842
FPPS	1	1.69	0.35	0.45223
FYB1	1	0.41	0.35	0.451
RHG25	1	0.71	0.36	0.44855
AK1C3	3	0.62	0.36	0.44855
DRB5	2	1.51	0.36	0.44249
ITB2	3	1.53	0.36	0.4389
RS26	2	0.65	0.37	0.43533
CLIC1	12	0.82	0.37	0.43533
NNRE	1	0.66	0.37	0.43297
RL15	1	0.63	0.37	0.43297
THIO	5	1.25	0.37	0.43297

COMD2	1	1.44	0.37	0.4318
RBM3	1	0.56	0.37	0.4318
F120A	1	2.39	0.37	0.43063
CFAI	4	1.09	0.37	0.43063
G6PD	2	0.80	0.37	0.42829
MOES	43	0.82	0.38	0.42597
CAPR1	1	0.80	0.38	0.41908
RL7A	2	0.74	0.38	0.41908
KV315	2	1.40	0.38	0.41794
VPS29	1	0.66	0.38	0.4168
IGJ	8	1.28	0.38	0.41567
PSMD2	3	0.82	0.39	0.41454
PP1R7	1	0.68	0.39	0.41117
LV310	1	1.24	0.39	0.40894
RS2	2	0.81	0.39	0.40561
PTPRC	2	0.69	0.40	0.4023
PSB5	1	0.56	0.40	0.40121
UBP15	1	1.40	0.40	0.39903
KPYM	28	0.85	0.40	0.39903
KV401	2	1.52	0.40	0.39794
SAMP	1	1.14	0.40	0.39577
BIN2	4	0.77	0.40	0.39577
IMDH2	1	1.30	0.40	0.39469
JAML	1	0.68	0.40	0.39469
CO6	1	1.71	0.40	0.39362
UBE2K	1	0.61	0.41	0.39147
EF1B	3	0.79	0.41	0.38722
P85A	1	0.67	0.42	0.38091
ZCCHV	2	1.41	0.42	0.38091
SC23B	4	0.83	0.42	0.38091
HNRPF	5	0.84	0.42	0.38091
FAS	2	1.33	0.42	0.37469
RL19	2	0.75	0.42	0.37469
PPIB	7	0.82	0.42	0.37469
RAVR1	1	0.62	0.42	0.37366
RS17	3	0.75	0.42	0.37366
ALBU_BOVIN	95	1.08	0.42	0.37263
NASP	5	0.82	0.43	0.37059
ANT3	4	1.16	0.43	0.36552
PP1G	1	0.69	0.43	0.36351
LAMC1	3	0.78	0.44	0.36051
NUDC1	1	0.68	0.44	0.35952
IDHP	1	1.71	0.44	0.35853
TES	1	0.79	0.44	0.35754

PPIH	1	0.70	0.44	0.35458
S10A4	3	0.83	0.45	0.35164
UBE3A	1	0.70	0.45	0.35067
AB1IP	2	0.81	0.45	0.34486
PDIA6	4	0.81	0.46	0.33724
SPB9	12	0.78	0.47	0.33255
UBA6	1	0.49	0.47	0.33068
KVD11	2	1.52	0.47	0.32606
FA98B	2	0.71	0.48	0.32148
UBCP1	1	0.69	0.48	0.31695
FLNA	49	0.85	0.48	0.31695
TCOF	4	1.16	0.48	0.31515
TCTP	3	0.84	0.49	0.31247
EF1A1	19	1.13	0.49	0.31158
KV116	1	1.94	0.49	0.31069
RINI	10	0.86	0.49	0.30715
IF4A1	9	1.19	0.50	0.30364
RAP1A	1	0.82	0.50	0.3019
FETUA	1	1.22	0.50	0.29843
IREB2	1	0.75	0.50	0.29843
RALY	2	1.16	0.50	0.29757
PSB7	1	0.61	0.51	0.29585
MPRI	1	1.43	0.51	0.29328
APOH	1	1.09	0.51	0.29328
CREG1	1	1.20	0.51	0.29158
RS6	3	0.83	0.52	0.28735
KAP2	1	0.84	0.52	0.28567
ANXA2	16	0.90	0.52	0.28567
PSMD3	3	0.85	0.52	0.284
SF3A3	1	0.72	0.52	0.28316
EHD1	4	0.79	0.52	0.28067
ACAP1	1	0.47	0.53	0.27901
GARS	1	0.83	0.53	0.27737
MAP4	4	0.79	0.53	0.27737
MYO1E	1	0.82	0.53	0.27654
HV69D	1	1.30	0.53	0.27572
APT	1	0.83	0.54	0.27084
TRFL	3	0.89	0.54	0.26922
TBA1C	18	1.17	0.54	0.26841
DRA	1	1.28	0.54	0.26761
MY18A	3	0.79	0.54	0.26761
CYBP	2	0.85	0.54	0.2668
PCNA	5	0.82	0.54	0.2652
EMAL4	1	0.74	0.55	0.2636

ABC3C	1	1.41	0.55	0.26122
HV307	2	1.18	0.55	0.26043
A1BG	1	1.10	0.55	0.25727
SYTC	3	0.85	0.56	0.25259
SPSY	1	1.16	0.57	0.24718
RS25	2	0.79	0.57	0.24718
BIEA	2	1.21	0.57	0.24336
RS18	2	1.30	0.57	0.2426
IMB1	5	0.85	0.57	0.24185
SRSF7	2	0.86	0.57	0.24109
HNRPM	4	1.14	0.58	0.23958
CD44	1	0.78	0.58	0.23882
YTHD2	2	0.86	0.58	0.23882
HV118	1	0.73	0.58	0.23508
LV321	2	1.17	0.58	0.23508
A2AP	1	1.07	0.58	0.23359
SRSF3	1	0.86	0.58	0.23359
ACTN1	3	0.86	0.58	0.23359
IGHG3	6	0.87	0.58	0.23359
PSMD4	3	0.90	0.59	0.2321
NHRF1	1	1.33	0.59	0.22621
SRSF8	1	0.85	0.60	0.22113
ERP44	1	1.19	0.60	0.2204
HV321	2	1.27	0.60	0.21968
LV147	1	1.19	0.60	0.21896
RS5	4	0.92	0.60	0.21896
PCNP	1	1.18	0.61	0.21681
GBB2	2	0.86	0.61	0.21467
CO3A1	1	1.07	0.61	0.21325
IGHM	19	1.29	0.61	0.21325
DDI2	1	1.16	0.61	0.21183
METK2	3	0.93	0.62	0.21042
HV102	1	0.64	0.62	0.20691
GSH1	1	0.83	0.63	0.20412
RL24	2	0.84	0.63	0.20412
H2AZ	2	0.77	0.63	0.20273
SYAC	5	0.80	0.63	0.20273
TRIR	1	1.28	0.63	0.19928
IF4B	3	1.17	0.63	0.1986
ARI1	1	1.49	0.64	0.19723
DDX42	2	1.22	0.64	0.19723
RL37	1	1.39	0.64	0.1945
KVD24	1	1.24	0.64	0.19246
AOC3	1	0.89	0.65	0.18977

RL8	3	0.88	0.65	0.18709
MK14	1	0.82	0.65	0.18575
FIBB	1	0.90	0.66	0.18243
PDLI5	1	1.23	0.66	0.18177
RANG	2	1.11	0.66	0.18111
LV151	1	1.35	0.67	0.17718
PDIA4	10	1.11	0.67	0.17522
PK3CD	1	0.82	0.67	0.17393
SF3A2	1	0.82	0.68	0.1707
TETN	3	0.96	0.68	0.16877
S10A9	3	1.18	0.68	0.16813
THBG	2	1.07	0.68	0.16685
RL28	1	1.37	0.68	0.16558
IF5A1	4	0.92	0.69	0.16368
RLA2	4	0.89	0.69	0.16368
LASP1	2	0.87	0.69	0.16178
TBB5	23	1.11	0.69	0.16052
RL37A	2	1.19	0.69	0.15927
ICAL	3	0.89	0.69	0.15864
GAPR1	1	1.11	0.70	0.1549
TBB4B	1	0.90	0.70	0.1549
PICAL	1	0.86	0.70	0.15304
PSMD6	1	0.91	0.71	0.15181
NSF1C	2	0.92	0.71	0.15181
KV320	2	1.21	0.71	0.1512
LAMB1	1	0.85	0.71	0.14997
CLIC3	2	1.15	0.71	0.14935
CORO7	1	1.22	0.71	0.14874
DTD1	1	0.74	0.71	0.14874
IGHG2	11	1.12	0.71	0.14874
RTCB	1	0.88	0.71	0.14813
CSK	1	1.11	0.71	0.14752
PRS7	2	1.12	0.71	0.14752
ENPL	20	0.87	0.71	0.14752
S10AC	1	0.83	0.71	0.1463
HV374	4	1.16	0.71	0.1463
CAP7	1	1.21	0.72	0.14448
LEUK	1	0.87	0.72	0.14388
KCY	3	0.89	0.73	0.13966
ITIH4	5	0.97	0.73	0.13847
ACINU	1	1.30	0.73	0.13549
RECQ1	1	0.86	0.73	0.1343
PEDF	1	1.23	0.74	0.13312
ZC3H4	1	0.86	0.74	0.13312

KV139	3	1.32	0.74	0.13312
RUVB2	1	0.85	0.74	0.13194
RL14	1	0.89	0.74	0.12901
GIMA4	1	1.23	0.75	0.12784
THRB	1	1.15	0.75	0.12784
IGLL5	9	1.22	0.75	0.12784
PRDX3	1	1.10	0.76	0.12205
IGKC	7	1.12	0.76	0.11862
HV330	4	1.16	0.76	0.11748
RS19	5	1.09	0.76	0.11748
RL6	3	0.90	0.76	0.11691
CAN2	2	1.09	0.77	0.11634
EVL	3	1.10	0.77	0.11634
TYPH	11	0.93	0.77	0.11238
CLIC4	1	0.85	0.78	0.10958
TRI25	2	0.91	0.78	0.10846
ERAP1	6	1.17	0.78	0.10846
PUR9	4	0.94	0.79	0.10237
HV349	1	0.94	0.79	0.10182
RL10	2	0.92	0.79	0.10182
VNN1	1	0.93	0.80	0.09854
HV551	1	0.87	0.80	0.098
IPYR	3	1.06	0.80	0.09691
NCKPL	2	0.91	0.80	0.09528
KV105	1	1.16	0.80	0.09474
BIP	13	0.94	0.81	0.0942
NAMPT	7	1.04	0.81	0.08938
LAMA1	1	1.13	0.82	0.0846
VPS4B	2	1.09	0.82	0.08407
MDHM	2	0.97	0.82	0.08407
IGLC2	3	1.15	0.83	0.08302
TXND5	11	0.94	0.83	0.08249
KVD29	2	1.05	0.83	0.0804
RS24	1	0.92	0.83	0.07988
CO3	7	0.98	0.83	0.07988
TIF1B	1	0.88	0.84	0.07831
OAS1	1	0.87	0.84	0.07624
LSM6	1	0.88	0.84	0.07572
EFHD2	4	1.04	0.84	0.07572
ERF3B	1	1.06	0.84	0.0752
STMN1	2	1.06	0.84	0.07366
IGHA2	4	1.10	0.84	0.07366
SNX6	1	0.95	0.85	0.07212
PPID	1	0.92	0.85	0.07212

GANAB	4	0.95	0.85	0.07109
ALBU	29	0.95	0.85	0.07007
COTL1	10	1.04	0.86	0.06803
ARHG6	1	0.93	0.86	0.06753
SPB10	1	0.89	0.86	0.06753
RS14	3	1.05	0.86	0.06651
RS13	2	1.06	0.87	0.05948
CH10	1	1.04	0.87	0.05899
AHSA1	1	0.96	0.87	0.05849
RL27A	2	0.93	0.88	0.0575
ARHG1	1	0.93	0.89	0.05306
HPT	4	1.03	0.89	0.05306
ROA0	2	1.05	0.89	0.05257
HXK3	3	0.96	0.89	0.05257
VASP	10	0.97	0.89	0.05257
S10A8	2	1.05	0.89	0.05061
HEMO	20	0.99	0.89	0.04964
IGHD	3	1.12	0.89	0.04915
PLD4	4	1.04	0.90	0.04769
TFG	1	0.95	0.90	0.04479
BAF	1	0.96	0.91	0.04335
PPP5	1	1.07	0.91	0.04287
IGHA1	17	1.03	0.91	0.04239
DEF3	1	0.96	0.92	0.0381
AL1B1	1	1.07	0.92	0.03716
KAP0	2	1.03	0.92	0.03574
C1TC	2	1.03	0.92	0.03527
CD97	1	1.09	0.92	0.0348
RL13	5	0.97	0.92	0.03433
FIBG	1	1.02	0.93	0.03245
ANXA1	14	0.97	0.93	0.03198
RS15A	2	0.98	0.93	0.02965
IGHG4	2	0.94	0.93	0.02965
GNAI2	3	1.03	0.94	0.02641
SNX1	1	0.98	0.95	0.02411
SHBG	1	1.02	0.95	0.02365
PSD12	1	0.98	0.95	0.02182
ELNE	4	0.97	0.95	0.02182
DBNL	1	1.02	0.96	0.01728
RS9	1	0.97	0.96	0.01592
MNDA	7	1.02	0.96	0.01592
PRS6A	1	1.02	0.97	0.01457
APOC3	1	1.00	0.97	0.01457
IGHG1	10	0.98	0.97	0.01412

**HV43D**	**1**	**0.99**	**0.97**	**0.01323**
**AN32E**	**2**	**0.99**	**0.97**	**0.01323**
**PDCD5**	**1**	**1.01**	**0.98**	**0.01055**
**AMPL**	**14**	**0.99**	**0.98**	**0.01055**
**S10A6**	**3**	**1.00**	**0.98**	**0.00745**
**CALR**	**6**	**0.99**	**0.98**	**0.00745**
**HV439**	**3**	**0.99**	**0.98**	**0.007**
**ANXA7**	**3**	**0.99**	**0.99**	**0.00656**
**RAC2**	**3**	**1.00**	**0.99**	**0.00612**
**PRTN3**	**3**	**0.99**	**0.99**	**0.00612**
**HV601**	**1**	**1.01**	**0.99**	**0.00568**
**RL26**	**2**	**1.01**	**0.99**	**0.00524**
**RS27L**	**2**	**1.01**	**0.99**	**0.00524**
**ROA3**	**5**	**1.00**	**0.99**	**0.00524**
**TBA1B**	**3**	**1.00**	**0.99**	**0.0048**
**FRIH**	**4**	**1.00**	**0.99**	**0.00349**
**PERM**	**18**	**1.00**	**1.00**	**0.00218**

Table S2: Cell input and sequencing output of the scRNA-seq experiment.

Subject	*Ex vivo*	Flu 6hrs	Flu 24hrs
A	2759	2102	1871
B	2794	2758	2796
C	2535	2450	2206
Total Cells per Condition	8088	7310	6873
Mean reads per cell	55,375	58,215	62,944
Median genes per cell	2,670	2,786	3,758
Doublets	2152	(excluded from analysis)	
Ambiguous	151	(excluded from analysis)	

Table S3: Top 25 enriched genes in the pDC scRNA-seq clusters, by cluster

Gene	avg_logFC	p_val_adj
CLUSTER 0		
FCER1G	2.1024666	0
C12orf75	2.0461347	0
MS4A6A	1.7763833	0
PTPRE	1.6217105	0
TAGLN2	1.5492633	0
ALOX5AP	1.5456969	0
UCP2	1.5376786	0
RNASE6	1.5040914	0
ZFP36L2	1.4492112	0
CST3	1.4329711	0
DERL3	1.430853	0
PLD4	1.3906609	0
PPP1R14B	1.3824086	0
GZMB	1.3781429	0
UGCG	1.3781111	0
TPM2	1.3715611	0
CORO1A	1.3562062	0
CCDC50	1.3482743	0
APP	1.3357869	0
SERPINF1	1.3109063	0
SAMHD1	1.2736115	0
IRF2BP2	1.2652994	0
BCL11A	1.2436351	0
GAPT	1.2419787	0

Gene	avg_logFC	p_val_adj
CLUSTER 1		
IFNA2	3.549002	0
IFNA14	3.36561	0
IFNA6	3.331724	0
IFNB1	3.189481	0
IFNW1	3.158891	0
IFNA17	3.157789	0
IFNA10	3.105879	0
IFNA8	3.069484	0
IFNA5	3.052137	0
IFNA4	2.975805	0
IFNA21	2.94575	0
IFNA1	2.645179	0
IFNA16	2.627565	0
IFNA7	2.594752	0
IFNL1	2.532747	0
PPP1R15A	2.506891	0
PUS10	2.17797	0
IL12A	2.163395	0
CCL3	2.132322	0
CCL4L2	2.089998	0
BCL2A1	2.05844	0
CCL4	2.050363	0
TNF	1.927204	0
NR4A3	1.847532	0

Gene	avg_logFC	p_val_adj
CLUSTER 2		
CCL4	1.702038	0
NCF1	1.598255	0
CCL4L2	1.555877	0
TNF	1.550849	0
NABP1	1.539063	0
HSPA1A	1.527175	0
CD83	1.503993	0
LTB	1.499888	0
HSPA1B	1.280324	0
IFIT2	1.265776	0
CCL3	1.229196	0
CDKN1A	1.226971	0
LTA	1.207946	0
CD40	1.157032	0
LDLRAD4	1.143193	0
HSP90AA1	1.099246	0
CCND2	1.086032	0
TYW3	1.013196	0
RGS1	1.003052	5.5E-284
PPP1R15A	0.996728	0
CALCRL	0.986798	5.6E-192
ATF3	0.967541	1.5E-222
HSPA8	0.92811	0
CCL3L1	0.924779	1.5E-108

Gene	avg_logFC	p_val_adj
CLUSTER 3		
IFI27	1.774985	0
PNOC	1.45832	5.1E-236
TNFSF10	1.341827	0
NEAT1	1.303959	0
IFI6	1.268901	0
CXCL13	1.261145	3.4E-165
FTL	1.205017	0
DUSP5	1.184991	0
CLEC2D	1.167247	0
HIST1H1C	1.135166	0
GAPDH	1.131192	0
ATOX1	1.07544	0
LY6E	1.022173	0
ISG15	1.007866	0
CD38	0.953347	0
SCT	0.948665	1.4E-102
STAT1	0.940203	0
COX5A	0.911369	0
PRDM1	0.8714	0
CASP3	0.869088	0
RSAD2	0.846212	0
SAT1	0.824952	1.9E-293
P2RY10	0.80955	0
TRIB1	0.804212	0

Gene	avg_logFC	p_val_adj
**CLUSTER 4**		
CD44	1.59138	0
CXCL10	1.428058	0
NMB	1.424394	1.79E-88
LTA	1.221621	0
CXCL11	1.20654	5.9E-127
CD40	1.09721	0
CCL4L2	1.073064	0
SLAMF7	1.063316	0
ID2	1.058091	0
CD83	1.049015	0
CALCRL	1.02762	0
FAM129A	1.025988	0
HSPA1A	1.025384	0
ALCAM	1.018413	0
REL	1.003306	2.5E-303
BCL2A1	0.999812	0
MIR155HG	0.986837	0
TFEC	0.926733	0
CFLAR	0.89305	0
BCL2L1	0.891324	0
CCL4	0.881024	0
NFKB1	0.859435	0
HERC5	0.85307	0
C15orf48	0.843186	1.7E-233

Gene	avg_logFC	p_val_adj
**CLUSTER 5**		
CXCL13	1.800695	0
CCR7	1.710081	0
TMSB4X	1.693879	0
TUBA1A	1.626864	0
SOX4	1.622532	0
BASP1	1.596734	0
FSCN1	1.446334	0
ZFP36L1	1.398223	0
CD70	1.386201	0
IFI27	1.374846	0
LMNB1	1.326046	0
MARCKS	1.299956	0
IFITM3	1.277995	0
CKB	1.273303	0
LY6E	1.206548	0
GAPDH	1.191684	0
TUBB	1.163445	0
SERPINB1	1.158247	0
RAB9A	1.135876	0
S100A11	1.119249	0
PKM	1.11292	0
CLEC2D	1.112227	0
BID	1.1038	0
TNFSF4	1.0764	0

Gene	avg_logFC	p_val_adj
CLUSTER 6		
MT2A	1.32218	0
EAF2	1.144386	0
GAPDH	1.141306	0
TPI1	1.079615	0
CCR7	1.077027	0
NEAT1	1.016605	0
LDHA	0.976995	0
CLEC2D	0.951816	0
HIST1H1C	0.939304	0
CLEC2B	0.921002	0
MPC2	0.910105	0
TALDO1	0.907717	0
GBP1	0.902111	0
UCHL1	0.899496	0
SPATS2L	0.898494	0
CSRP2	0.898457	0
CMTM7	0.880067	0
PKM	0.879726	0
IFIT1	0.865848	0
IFI27	0.845355	0
CTSC	0.844856	4.2E-275
PRDX1	0.841203	0
FTL	0.839755	0
TYMP	0.829577	0

Gene	avg_logFC	p_val_adj
CLUSTER 7		
LTB	1.369874	0
TYW3	1.01182	0
HSPA1A	0.995985	0
BTG1	0.91905	0
HSPA8	0.870512	0
BBX	0.860342	0
LAP3	0.833926	0
DCPS	0.802439	2.1E-250
CSF2RB	0.795061	3.1E-300
PAG1	0.779625	0
HSP90AA1	0.77947	0
SMC6	0.772563	0
FYB1	0.769351	1.7E-303
SLC12A2	0.768546	5.4E-266
EIF2AK2	0.762227	0
SERPING1	0.759044	0
CRYZ	0.751272	0
TSPAN13	0.745545	0
TCL1A	0.738809	4.82E-61
UBE2L6	0.738395	0
PIM3	0.734997	0
SAMD9	0.730834	0
DDX60	0.725135	0
DNASE1L3	0.721615	0

Gene	avg_logFC	p_val_adj
CLUSTER 8		
CCND2	1.643757	0
KHK	1.294032	7.8E-208
CCL19	1.163064	2.12E-94
MYBL2	1.13259	0
BTG1	1.108356	0
ERICH3	0.859103	5.5E-185
TCL1A	0.827502	3.87E-95
CALR	0.774469	0
NLRP7	0.764753	0
NKG7	0.731817	5.4E-129
SYNGR2	0.716912	1.2E-235
IFI27	0.712686	2.3E-159
CTSZ	0.699129	7.1E-223
GPX4	0.699015	2.3E-254
HSP90B1	0.692583	1.1E-255
WNT10A	0.688987	5.9E-284
TXN	0.686913	2.3E-146
PNOC	0.64691	9.77E-46
COX5A	0.646351	3.3E-268
SCT	0.643599	8.3E-114
VOPP1	0.626633	1.4E-234
BTG2	0.621961	3.7E-132
P2RY6	0.616117	6.4E-221
IGHM	0.61362	2.1E-278

Gene	avg_logFC	p_val_adj
CLUSTER 9		
CXCL11	1.83922	2.1E-253
RSAD2	1.697238	3.1E-295
CXCL10	1.634519	1.1E-204
HSPA1A	1.127474	1.9E-192
BTG1	1.092461	1.5E-159
HMOX1	1.081575	1.58E-61
PARP14	1.023822	2.5E-245
TFEC	1.014234	1.3E-215
AKR1C3	0.996355	2.2E-153
EMP3	0.990186	1.9E-197
ENDOG	0.962219	3.93E-94
CSF2RB	0.949516	2.4E-141
RNF213	0.916677	1.2E-224
HSPA1B	0.894641	1E-132
OAS3	0.883837	2.9E-206
HMGCS1	0.883055	2E-128
CXorf21	0.87998	2.25E-81
SQSTM1	0.830337	1.4E-103
MX1	0.810703	9.9E-192
PIM3	0.808803	3.3E-166
CMPK2	0.801006	3.9E-189
LAP3	0.798204	2.1E-202
DENND1B	0.794405	6.4E-187
CXCL9	0.794203	1.8E-75

Gene	avg_logFC	p_val_adj
CLUSTER 10		
MALAT1	1.213465	2.18E-65
XIST	0.930008	3.13E-83
NEAT1	0.816518	4.92E-39
N4BP2L2	0.689664	3.67E-62
MT-ND5	0.657111	8.48E-56
POLR2J3.1	0.648099	6.96E-39
CHD9	0.622404	3.93E-35
DDX17	0.613559	4.82E-50
MT-CO1	0.580231	7.42E-23
PNISR	0.579236	5.13E-43
HNRNPH1	0.566843	8.18E-44
MT-ND2	0.55709	1.23E-51
ARID1B	0.551148	1.61E-37
ANKRD12	0.545767	1.94E-39
RSRP1	0.543745	1.48E-35
FTX	0.541207	2.98E-36
RBM39	0.532532	1.34E-39
PNN	0.525924	5.7E-32
RNF213	0.522187	8.38E-23
ZNF207	0.521197	1.74E-37
CCNL1	0.519185	7.36E-32
INTS6	0.518957	4.69E-43
ZEB2	0.517619	7.99E-31
CCDC88A	0.510893	1.71E-35

Gene	avg_logFC	p_val_adj
CLUSTER 11		
IFNA1	3.23627	4.3E-270
IFNA16	3.209599	3.2E-243
CYTOR	3.129515	4.2E-286
IFNA4	2.93449	1.8E-241
IFNA21	2.841742	1.1E-259
IFNL1	2.831011	7E-175
IFNA8	2.686882	1.4E-227
IFNA17	2.634333	9.1E-205
IFNA5	2.622508	6.6E-286
MIR4435-2HG	2.60344	0
CCL5	2.537599	1.1E-156
IFNW1	2.459461	3E-239
IFNB1	2.435702	2.8E-228
IFNA10	2.431466	2.5E-222
GPR34	2.346484	0
IFNA6	2.338089	4.1E-118
DKK1	2.331726	0
CXCL8	2.218949	1.8E-295
IFNA14	2.205407	2.6E-209
IFNA7	2.200717	0
IFNE	2.190019	0
CCL3L1	2.161842	2.3E-186
BET1	2.15487	5.1E-254
CCL3	2.075043	4.2E-173

Gene	avg_logFC	p_val_adj
CLUSTER 12		
TMEM267	1.622947	7.6E-122
CTSC	1.412497	7.8E-144
DUSP11	1.365048	9.2E-156
IL12A	1.318169	1.9E-135
GRSF1	1.187564	1.2E-157
PNOC	1.167879	1.3E-42
CCR7	1.152894	2.5E-126
RGS1	1.14512	7.33E-48
IFI27	1.137594	1.1E-107
IGFBP4	1.092764	1.2E-146
HARS	1.083338	3.1E-105
MPC2	1.05995	6.7E-112
CMTM7	1.050202	1.4E-139
CLECL1	1.034513	1.6E-121
CAB39L	1.028687	1.8E-197
MARCKSL1	1.024607	1.8E-80
RNF115	1.019902	7.58E-82
GPR34	0.960441	9.67E-57
CD48	0.943073	1.59E-40
GPR160	0.934233	2.3E-174
CLEC2D	0.919263	7.5E-106
REL	0.917475	2E-110
CRYBG1	0.900703	2.1E-206
ENPP2	0.898065	1.41E-87

Gene	avg_logFC	p_val_adj
CLUSTER 13		
IGHA1	6.122379	2.6E-143
IGLC3	4.976468	1
IGKC	4.781452	1.06E-07
IGHG2	4.599817	7.8E-281
IGLC2	4.517642	1
IGHG4	4.310132	6.6E-148
IGHG1	4.141818	2.1E-167
IGLC7	4.047892	4.12E-33
IGHA2	3.653254	0
IGHM	3.340308	0.000234
IGHG3	3.121733	0
JCHAIN	2.643538	6.5E-102
CD79A	2.092111	0
IGLL5	1.981569	1.8E-283
CD27	1.936166	0
TNFRSF17	1.820993	3.1E-174
POU2AF1	1.777515	0
MZB1	1.743814	2.23E-98
FKBP11	1.612301	6.2E-152
KLF2	1.533383	0
TENT5C	1.450866	0
IGKV4-1	1.360235	0
PIM2	1.184434	1E-108
CD52	1.177831	3.4E-82

Gene	avg_logFC	p_val_adj
CLUSTER 14		
CCND2	1.673926	7.29E-63
PNOC	1.511161	2.7E-20
PCLAF	1.30334	0
TYMS	1.298088	0
PCNA	1.036432	5.1E-85
IFI27	0.984043	8.59E-26
CLSPN	0.973058	0
MCM4	0.956267	0
CEP55	0.922884	2.16E-97
NASP	0.870973	7.03E-52
GAPDH	0.867348	6.01E-34
GINS2	0.862674	0
CKS1B	0.852369	1.79E-49
EDNRB	0.845541	4.69E-40
SCT	0.840879	2.31E-13
CTSC	0.829415	8.46E-18
MCM7	0.811443	3E-107
CHEK1	0.807757	0
CLECL1	0.802669	4.91E-28
H2AFZ	0.802205	5.25E-33
IGHM	0.793891	2.11E-34
MCM3	0.789192	6.32E-61
COX5A	0.781364	8.36E-40
TUBA1B	0.781008	8.57E-40

Gene	avg_logFC	p_val_adj
CLUSTER 15		
PLCG2	4.288975	8.08E-58
HEXIM1	2.578878	1.11E-60
JUN	2.402232	4.64E-42
GADD45B	2.249662	1.04E-45
IER3	2.245483	1.09E-27
NFKBIA	2.214461	7.69E-24
SNHG12	2.176202	2.27E-83
INTS6	2.146815	1.84E-36
IER5	2.109135	8.03E-45
RASD1	2.051184	4.96E-67
IER2	1.985599	1.69E-29
MAFB	1.953123	0
FOS	1.934448	3.7E-144
SRSF7	1.919737	4.55E-39
PMAIP1	1.84026	6.3E-29
SOCS1	1.80901	2.87E-43
EGR1	1.769766	4.5E-264
ID2	1.763281	3.44E-36
PLK2	1.762039	2.1E-301
DNAJB1	1.74626	1.44E-05
TUBB4B	1.724608	1.26E-27
JUNB	1.68239	5.9E-39
CXCL8	1.644674	0.524263
HSPA1B	1.624778	3.9E-31

Gene	avg_logFC	p_val_adj
CLUSTER 16		
GIMAP7	1.851219	0
IL7R	1.678736	1.7E-105
TRBC2	1.656116	0
GZMK	1.640609	0
CD79A	1.640104	5.53E-14
IGLC3	1.604881	1
GIMAP4	1.549939	0
MT2A	1.48322	7.18E-14
EVL	1.457164	5.3E-103
LIMD2	1.346851	7.97E-26
KLF2	1.333666	2.7E-184
IL32	1.263929	0
TRAC	1.210002	0
TRBC1	1.201439	0
CD52	1.186019	5.96E-22
CXCR4	1.185677	2.12E-08
AC245297.3	1.154835	1.72E-48
ITM2B	1.146894	4.57E-30
CD3D	1.11465	0
TRDC	1.091593	1.2E-119
GBP1	1.084558	1.07E-25
CD48	1.049761	1.29E-24
GBP4	1.045723	3.16E-19
SMCHD1	1.031087	4.5E-18

Gene	avg_logFC	p_val_adj
CLUSTER 17		
LYZ	3.203738	0
S100A10	2.288291	5.96E-20
S100A4	2.078156	9.89E-17
COTL1	1.854883	1.88E-53
CST3	1.586403	2.32E-15
PPP1R14A	1.262968	9.48E-31
FOS	1.190144	7.8E-125
FGL2	1.179553	1.1E-104
AXL	1.130974	3.69E-93
AIF1	1.109946	1.34E-13
ITGB2	1.085394	3.83E-20
FCGRT	1.077133	3.17E-14
CORO1A	1.076163	6.27E-12
ANXA1	1.025688	3.08E-42
TXNIP	1.021241	4.54E-10
VIM	1.016814	1.78E-08
OTULINL	0.992976	6.86E-19
SAMHD1	0.974915	1.7E-10
ACTG1	0.964491	8.87E-09
S100A6	0.951039	5.65E-10
DAB2	0.909922	8.88E-13
CCND3	0.885548	4.27E-07
LIMD2	0.877811	6.07E-11
CTSH	0.87696	6.38E-10

Table S4: GaP Subject Demographics

Subject ID	Age	Sex:	BMI:	Medical Conditions (self-reported)	Ethnicity
74178	35	Female	22.04	Drug Allergies, Wears Glasses	Caucasian
74315	34	Female	27.43	Asthma, Seasonal Allergies, Urinary Tract Infections (UTI)	Caucasian
76746	27	Female	20.25	Asthma, Gastroesophageal Reflux Disease (GERD), Wears Glasses, Celiac Disease	Caucasian
